# From Polymers to Rings and Back Again: Chemical Recycling of Polyesters to Macrolactones

**DOI:** 10.1002/anie.202423478

**Published:** 2025-03-27

**Authors:** Madeleine L. Smith, Thomas M. McGuire, Ryan W. F. Kerr, Charlotte K. Williams

**Affiliations:** ^1^ Department of Chemistry Chemistry Research Laboratory University of Oxford 12 Mansfield Rd Oxford OX1 3TA UK

**Keywords:** Catalysis, Chemical recycling, Depolymerization, Lactones, Polyesters

## Abstract

Cyclic anhydride and epoxide ring‐opening copolymerization is a versatile and controlled route to make polyesters, gaining attention in different application sectors. But so far, the chemical recycling of these polyesters to cyclic monomers is under‐explored. Here, the catalytic chemical recycling of aliphatic polyesters to selectively form 16‐ and 18‐membered lactones is presented. The recycling reactions are catalyzed using commercial tin(II) octoate and conducted in the polymer melt (230 °C) resulting in high conversions to the macrolactones (>90%). The recycled macrolactones undergo catalyzed ring‐opening polymerizations to produce polyesters with equivalent properties to the virgin materials.

## Graphical abstract

Here, the chemical recycling of polyesters derived from the ring‐opening copolymerization of cyclic anhydride with epoxide is presented. The polyesters are selectively depolymerized using commercial tin(II) octoate to form 16‐ and 18‐membered lactones with high selectivity and conversions. The recycled macrolactones undergo catalyzed ring‐opening polymerizations to produce polyesters with equivalent properties to the virgin materials.

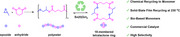

Polymer chemical recycling is essential for the future circular plastics economy.^[^ ^1^, ^2^, ^3^, ^4^ ^]^ It both reduces demand for virgin raw materials and saves on the greenhouse gas emissions associated with monomer production while also avoiding the material degradation often associated with mechanical recycling.^[^ ^5^, ^6^, ^7^, ^8^ ^]^ For chemical recycling to be technologically feasible, polymers should have accessible monomer–polymer equilibria.^[^ ^9^ ^]^ Oxygenated polymers, such as polyesters, polycarbonates, and polyethers, benefit from lower temperature equilibria and are, therefore, excellent candidates for chemical recycling.^[^ ^5^, ^10^, ^11^ ^]^

An important method to produce polyesters is the ring‐opening copolymerization (ROCOP) of cyclic anhydrides and epoxides.^[^ ^12^, ^13^, ^14^, ^15^, ^16^ ^]^ The reaction requires a catalyst and, in the best cases, is fast, selective, and very well controlled, delivering polyesters with predictable molar mass/narrow dispersity.^[^ ^12^, ^14^, ^17^, ^18^, ^19^ ^]^ The best catalysts are highly effective at low loading and can selectively polymerize a wide range of commercial, functionalized, and bio‐derived monomers. The ROCOP processes typically occur at 50–150 °C, which are significantly lower temperatures than applied in diol/diacid polycondensations.^[^ ^17^, ^18^, ^20^, ^21^, ^22^ ^]^ Further, ROCOP can be used for the synthesis of novel architectures and/or block polymers. Low molar mass polyester polyols, produced by epoxide/anhydride ROCOP, are important precursors to higher polymers, for example, polyurethanes or polyester resins. Alternatively, higher molar mass ROCOP‐polyesters are high strength engineering plastics, elastomers, and adhesives.^[^ ^23^, ^24^ ^]^ Given the range of applications accessed by these ROCOP‐derived polyesters, it is also important to consider their end‐life recycling options.

Currently, there are no direct routes for the closed loop chemical recycling of ROCOP‐derived polyesters to their monomers. On the other hand, there is track record for the depolymerization of similar aliphatic polyesters produced by diol/diacid polycondensation. As early as the 1930s, Carothers and coworkers reported simple catalysts, for example, Zn(II) or Mg(II) salts, which depolymerized aliphatic polyesters, produced by condensation methods, to form lactones; in some cases, dimeric or higher order cyclic esters were formed.^[^ ^25^, ^26^ ^]^ The polyester repeat unit length influenced the relative stability of the lactone formed, with macrolactones being stable for ring sizes ≥12. The investigation revealed that as the ring size of the monomeric macrolactone lactone increased, so the selectivity for its formation increased (versus dimeric or higher order cyclic esters). Once the lactone ring size exceeded 15, the monomeric macrolactone was exclusively formed. The catalyzed depolymerization of other condensation polyesters, such as poly(terphthalates) or poly(ethylene furanoates), required high dilution conditions and typically formed product mixtures including different cyclic and linear oligoesters.^[^ ^27^, ^28^, ^29^ ^]^ Very recently, Chen and coworkers reported on the recycling of propionate poly(hydoxyalkoanoates), another class of aliphatic polyesters, to form 4‐ or 12‐membered lactones.^[^ ^30^ ^]^ In agreement with Carothers’ earlier work, lactone mixtures were always obtained. The alternative polyester hydrolysis to diols and diacids is, of course, very well‐known but significant energy and additional chemical processes are required to produce epoxides and anhydrides from these raw materials.^[^ ^11^, ^31^, ^32^ ^]^

When considering the optimum conditions for ROCOP‐polyester chemical recycling, it is important to form a lactone suitable for repolymerization directly without requiring further processes. In addition, any recycling process should occur at low temperatures, with minimal catalyst and without requiring high vacuum or gas flow rates (to drive reactions).

The development of efficient, catalyzed depolymerizations operating in neat polymer melts are a priority as solvents increase expense and greenhouse gas emissions, as well as introducing separation steps.^[^ ^33^, ^34^, ^35^ ^]^The depolymerization thermodynamics of ROCOP‐derived polyesters do not favor the direct reformation of the epoxide and anhydride monomers. Inspired by Carothers seminal work,^[^ ^25^, ^26^ ^]^ we hypothesized an alternative recycling route where the ROCOP‐derived polyesters are depolymerized to macrolactones, which when combined with a suitable catalyst, undergo ring‐opening polymerization to re‐form the polyesters (Figure 1).

**Figure 1:**
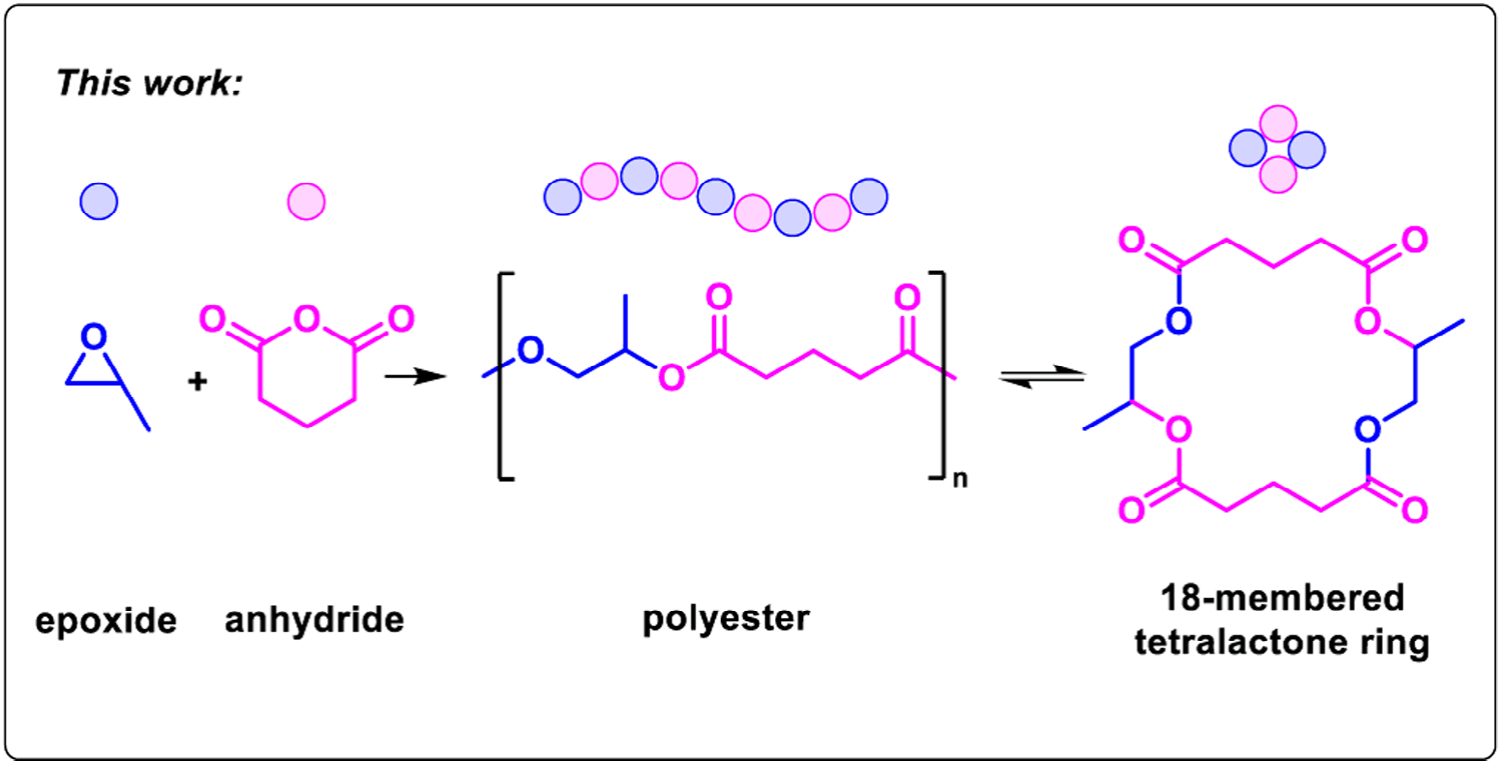
Catalyzed chemical recycling of poly(PO‐*alt*‐GA) to an 18‐membered ring macrolactone (note the other regio‐isomer is illustrated in Fig. 2).

The investigation of ROCOP‐derived polyester recycling was undertaken using the polymer produced from propene oxide and glutaric anhydride as these monomers are commonly studied. To evaluate its recyclability, α‐benzyl alcohol‐ω‐hydroxy poly(propylene oxide‐*alt*‐glutaric anhydride) (poly(PO‐*alt*‐GA)) was prepared by ROCOP of PO and GA. The ROCOP was performed using a phosphazene base, P_2_‐*^t^* Bu, and resulted in a regiorandom polyester as evidenced by NMR spectroscopy (*M*_n_, _SEC_ = 4800 g mol^−1^, *Đ* = 1.83, see Supporting Information for details). The polymer was purified by stirring over Amberchrom 50WX8 and silica chromatography. It was stable up to >300 °C, which is consistent with complete removal of all catalyst residues. To monitor its recycling, the depolymerization reactions were monitored by thermal gravimetric analyses (TGA). We, and others, have developed methods to evaluate variable and isothermal mass loss versus time data (from TGA), which when combined with analysis of the small‐molecule products, provides insight into both the rate and selectivity of polymer chemical recycling.^[^ ^33^, ^34^, ^35^, ^36^ ^]^ A range of metal catalysts were evaluated for the chemical recycling, including Ca(II)Oct_2_, Ba(II)Oct_2_, Zn(II)Oct_2_, Zr(IV)Oct_4_, Bi(III)Oct_3_, Sn(II)Oct_2_, Sn(IV)Oct_4_, and Mg(II)Cl_2_ (see Supporting Information for details). In these experiments, the control polymer (without catalyst) showed the on‐set of mass loss % at 300 °C, whereas the different catalysts reduced both the on‐set temperature and increased the rates of mass loss. Of the catalysts tested, Sn(II)Oct_2_ resulted in the greatest polymer mass loss (61%), indicating it was the most efficient (Figure S13). It also significantly reduced the polymer recycling onset temperature to 216 °C (Figure 2, *T*_d,5% _= 313 °C for poly(PO‐*alt*‐GA)).

**Figure 2:**
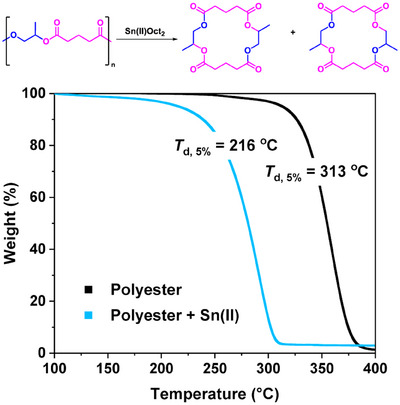
Mass loss (weight) versus temperature illustrating the catalyzed depolymerization of poly(PO‐*alt*‐GA) (blue data with Sn(II)(Oct)_2_) compared against the uncatalyzed reaction (black data). The reactions were conducted under N_2_ flows of 25 mL min^−1^, heat ramp 2 °C min^−1^, [catalyst]_0_:[poly(PO‐*alt*‐GA)]_0_ 1:500.

To determine the recycling activation energy, poly(PO‐*alt*‐GA) was depolymerized under different heating rates, with and without Sn(II)Oct_2_, and Flynn–Wall analysis was applied (see Supporting Information for details).^[^ ^37^ ^]^ The reaction where Sn(II)Oct_2_ was present had a significantly lower activation energy of 110 kJ mol^−1^ compared with the value of 260 kJ mol^−1^ for the control depolymerization reaction.

To characterize the products of the reaction, the depolymerization was performed on a larger scale using a Kugelrohr distillation apparatus (see Supporting Information for details). A clear, highly viscous product that solidified on cooling was collected. Poly(PO‐*alt*‐GA) conversions of 70% were achieved within 3 h and, in all cases, >99% mass was conserved. Analysis of the product by NMR spectroscopy, GC‐MS, and X‐ray crystallography identified an 18‐membered tetralactone cyclic molecule comprising of two repeat units of GA and PO (Figure 3). All the different isomers of the tetralactone were formed, as evidenced by both NMR spectroscopy and by analysis of the crystals that showed the E regioisomers (Figures S14–18).^[^ ^38^ ^]^ This reflects the regiorandom nature of the starting polyester. The tetralactone was crystalline and had two melt temperatures at 74 °C and 93 °C, which could be consistent with both diastereo‐ or regioisomer pairs. The depolymerization kinetics were evaluated at 230 °C, and it is essential to appreciate that no reaction occurs at this temperature without the catalyst being present. The mass loss data were fit to an exponential that indicates the reaction has a first order dependence on polymer mass. The order in catalyst was determined using an initial rates method (see Supporting Information for further details). The plot of the natural log of the rate coefficient versus the natural log of the catalyst concentration showed a gradient of 0.762.

The data are interpreted by some catalyst dimerization occurring under the high loadings used in these experiments; indeed, others have noted the tendency for related Sn(II) complexes to dimerize at high concentrations.^[^ ^39^ ^]^ The depolymerization may proceed via either random chain scission or a chain‐end catalyzed series of reactions or mechanisms. To distinguish between the two, a sample of acetyl end‐capped poly(PO‐*alt*‐GA) was subjected to identical catalyzed recycling conditions.

**Figure 3:**
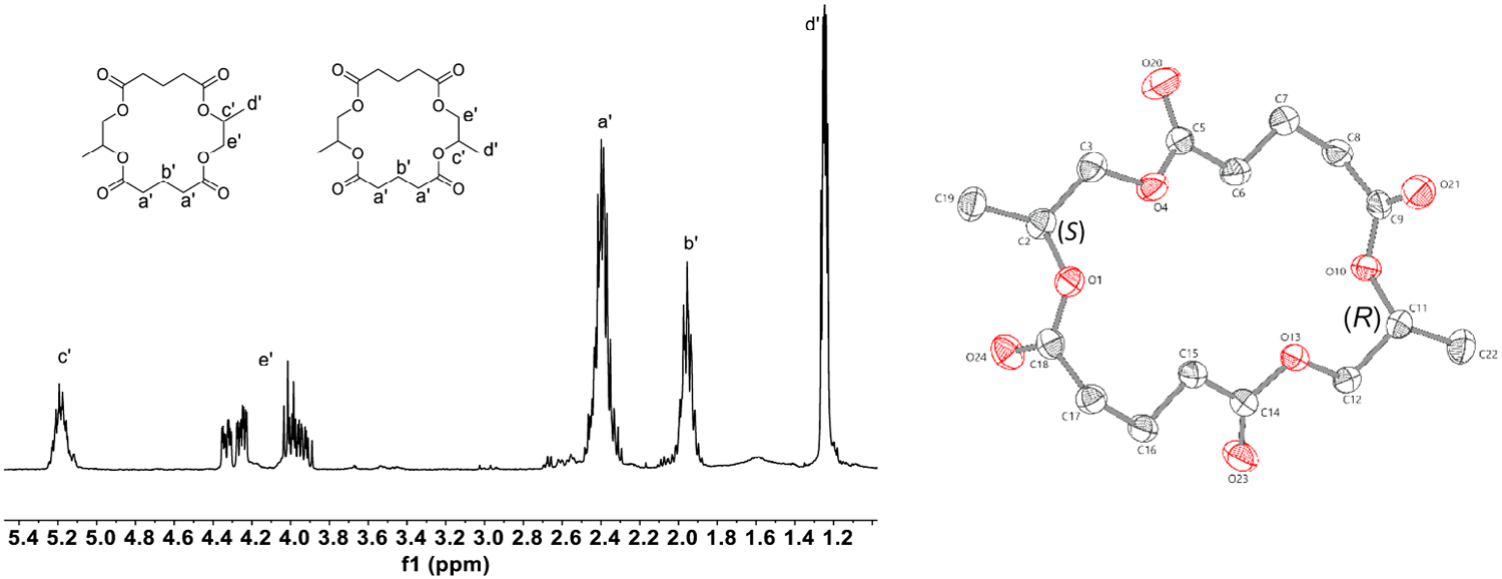
Characterization data for the macrolactone formed by recycling of poly(PO‐*alt*‐GA). LHS: ^1^H NMR spectrum (CDCl_3_) and RHS: molecular structure determined by single crystal X‐ray diffraction of the E‐trans tetralactone (Note: one enantiomer is shown but a racemic mixture is observed in the crystal, see Supporting Information). Depolymerization conducted at 230 °C, [Sn(II)Oct_2_]_0_:[poly(PO‐*alt*‐GA)]_0_ 1:10, 5 mbar vacuum.

The end‐capped polymer showed a depolymerization rate constant (*k_obs_*) only slightly lower than that observed for the dihydroxyl end‐capped polymer (Table 1, Entry 6). These findings suggest that macrolactone formation occurs by a random chain scission mechanism.

**Table 1:** Kinetic data for the depolymerization of poly(PO‐*alt*‐GA) using Sn(II)Oct_2_ catalyst.^a)^

Entry	[Sn]_0_/[Polyester]_0_	TON^b)^	Activity (h^−1^)^c)^	Rate Constant (h^−1^)^d)^	Conv. After 10 h (%)^e)^
1	1:10	9	5.6 ± 0.5	0.55 ± 0.05	90 ± 1
2	1:50	46	8.3 ± 0.1	0.18 ± 0.004	91 ± 6
3	1:100	74	8.2 ± 0.6	0.093 ± 0.007	74 ± 3
4	1:500	150	14 ± 1.7	0.033 ± 0.004	30 ± 3
5^f)^	1:100	71	7.2 ± 0.4	0.082 ± 0.005	71 ± 5
6^g)^	1:10	10	3.1 ± 0.5	0.33 ± 0.007	95 ± 3

^a)^ Depolymerizations performed under N_2_ flow rate 25 mL min^−1^, 230 °C, [catalyst]_0_:[poly(PO‐*alt*‐GA)]_0_ 1:10–500.

^b)^ Productivity or TON = (moles of epoxide converted)/(moles of catalyst), all values reported after 10 h.

^c)^ Activity or TOF = (moles of poly(PO‐*alt*‐GA) consumed from 5% to 20% conversion/moles of catalyst/time.

^d)^ *k*_obs_ determined from the linear fit of the ln[(sample weight)_t_/(sample weight)_0_] versus time plot between 95% and 80% weight.

^e)^ Poly(PO‐*alt*‐GA) conversion corresponds to the total weight loss of poly(PO‐*alt*‐GA)] after 10 h.

^f)^ Depolymerization using Sn(II)(O*^n^* Bu)_2_.

^g)^ Depolymerization using acetyl end‐capped poly(PO‐*alt*‐GA).

Depolymerizations were also conducted using a homoleptic tin alkoxide complex, Sn(II)(O*^n^* Bu)_2_, and these showed equivalent rates to experiments conducted, under otherwise identical conditions, using Sn(II)Oct_2_ (Table 1, Entry 5). These data suggest the two complexes feature the same (or very similar) active sites. The active site is proposed to be a Sn(II)‐alkoxide intermediate that reacts with the polyester chain via intermolecular transesterifications to form the thermodynamically stable 18‐membered ring.^[^ ^35^ ^]^

The recycling reaction selectivity was >99%, as measured by GC‐MS, for the 18‐membered tetralactone ring. Such high selectivity is beneficial and to understand the factors underpinning it, DFT calculations were undertaken to compare 18 versus 9 or 27‐membered lactones. These calculations reveal that all isomers of the 18‐membered tetralactone have a lower enthalpy (by ∼11 kcal) and lower Gibbs free energy (by ∼6 kcal) than those of the 9‐membered dilactone. In addition, the calculations show that the formation of the tetralactone ring is significantly more favorable than reforming the epoxide and anhydride monomers (Figure S35 and Table S3). The higher stability of larger macrolactone ring sizes (>12) compared to intermediate ring sizes (7–11) is proposed to arise from higher transannular strain during the cyclization of intermediate ring sizes.^[^ ^40^, ^41^ ^]^ As ring size increases, the ring strain becomes insignificant and cyclization is more thermodynamically favorable. The calculated Gibbs free energy of the 27‐membered hexalactone is very similar to that of the 18‐membered tetralactone. However, the entropic penalty for cyclization increases with the incorporation of more atoms and, thus, formation of the 18‐membered lactone is favored.

To establish the utility for the tetralactone, formed by chemical recycling, it should be polymerizable. The ring‐opening polymerization (ROP) of related macrolactones is known and tends to be entropically driven; such processes are most successful at high temperatures as these favor both the thermodynamics and kinetics of polymerization.^[^ ^42^ ^]^ Accordingly, the polymerization of the tetralactone was tested using neat monomer, at 150 °C and using the same Sn(II)Oct_2_ as the catalyst, at a loading of [Sn(II)Oct_2_]_0_:[benzyl alcohol]_0_:[tetralactone]_0_ 1:1:50 (see Supporting Information for details). The macrolactone ROP was successful, if slow, reaching complete conversion of 95% and producing the same polymer structure as prior to the recycling.

The polymerization conversion versus time data fit exponential kinetics that is consistent with first order rate dependence on tetralactone concentration. The polymerizations are quite slow, showing *k*_obs_ of 0.100 h^−1^, but such values are quite typical for entropically driven ring‐opening polymerizations (Figure 4). It was encouraging that the polymerization equilibrium occurred at ∼95% conversion. The resulting polyester showed an identical chemical structure and molar mass to the starting polymer. For example, its ^1^H NMR spectrum and size exclusion chromatography (SEC) data were very close to those of the starting polymer (recycled poly(PO‐*alt*‐GA): *M*_n, SEC _ = 4700 g mol^−1^, *Đ* = 1.25).

**Figure 4:**
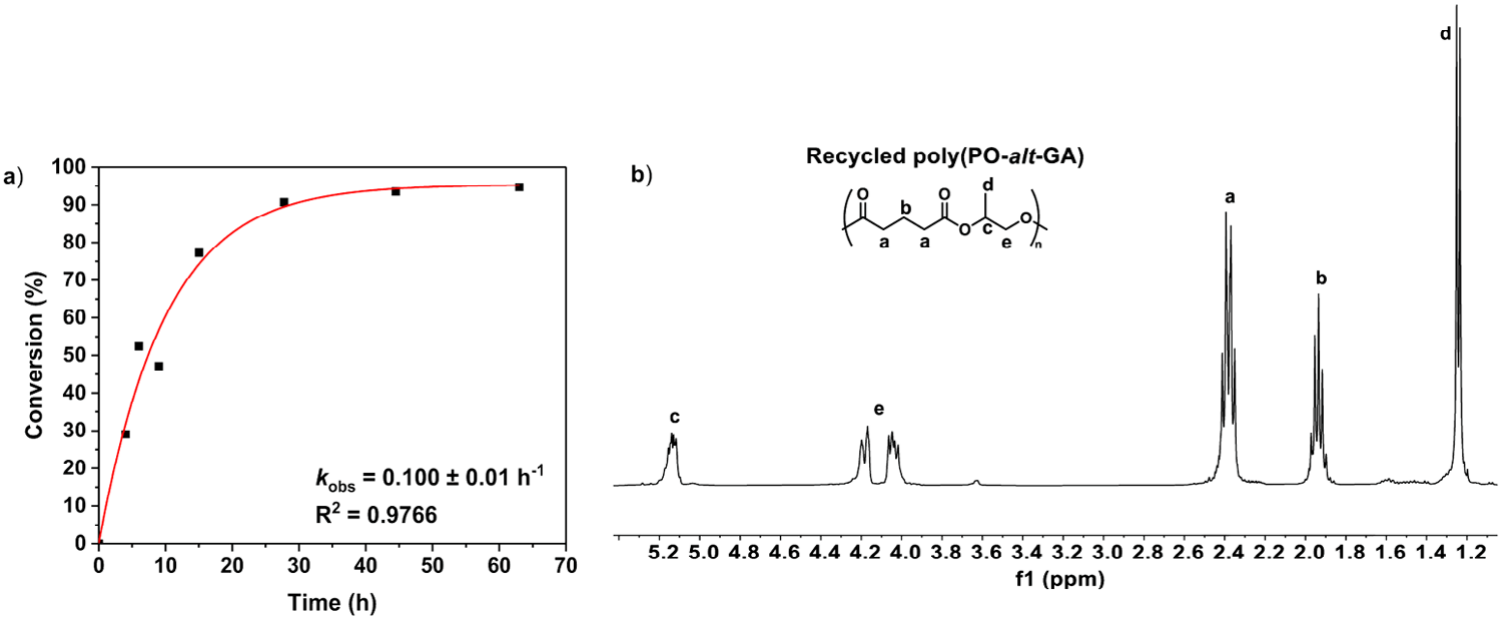
Formation of poly(Po‐alt‐GA) by the ROP of the macrolactone with Sn(II)Oct_2_ as the catalyst. a) Plot of polyester conversion versus time for the polymerization of the tetralactone (conversion: [Polyester] = 1‐[lactone]). The polymerization rate constant, *k*_obs_, was obtained from the exponential fit of the data. b) ^1^H NMR spectrum of the crude product, poly(PO‐*alt*‐GA). Polymerization conditions: 150 °C, [Sn(II)Oct_2_]_0_:[benzyl alcohol]_0_/[tetralactone]_0_ 1:1:50.

To explore the potential to apply the chemical recycling to other polyesters, two other ROCOP‐derived polyesters were investigated (Table 2). All the polyesters were stable when heated at 230 °C for 10 h, in the absence of catalyst. On mixing the polymers with Sn(II)Oct_2_, the catalyzed depolymerization temperature of poly(α‐butylene oxide‐*alt*‐glutaric anhydride) reduced from 314 to 238 °C and for poly(propylene oxide‐*alt*‐succinic anhydride) from 294 to 245 °C. The depolymerizations were all performed on a laboratory scale, using Kugelrohr distillation apparatus to isolate the lactones. In all cases, the reactions were highly selective in forming the tetralactone products comprising two repeat units of the ring‐opened anhydride and epoxide.

**Table 2:** Chemical Recycling of ROCOP‐derived polyesters to produce macrolactones.

Polymer	*T*_d,5%_ (°C)^a)^	Recycling Onset Temperature with Sn(II)Oct_2_ (°C)^b)^	Activity (h^−1^)^c)^	Rate Constant (h^−1^)^d)^	Conversion at 10 h (%)^e)^	Selectivity (%)^f)^	Depolymerization Product^g)^
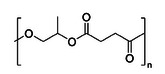 Poly(PO‐*alt*‐SA)	294	245	3.2 ± 0.3	0.35 ± 0.03	86 ± 1	>99	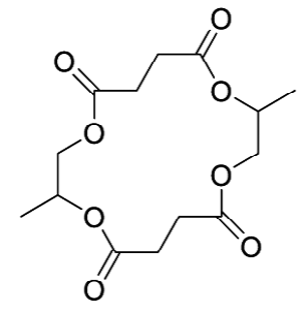
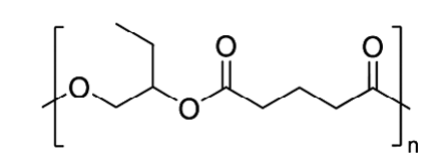 Poly(BO‐*alt*‐GA)	314	238	1.8 ± 0.02	0.19 ± 0.01	85 ± 1	74	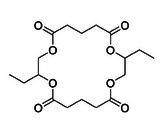

^a)^ Depolymerizations conducted under N_2_ flow rate 25 mL min^−1^, heat ramp 2 °C min^−1^.

^b)^ Depolymerizations conducted under N_2_ flow rate 25 mL min^−1^, heat ramp 2 °C min^−1^, [Sn(II)Oct_2_]_0_:[polyester]_0_ 1:500.

^c)^ Activity = TOF = (moles of polyester consumed (5%–20% conversion)/mole of catalyst/time. Depolymerizations performed under N_2_ flow rate 25 mL min^−1^, 230 °C, [catalyst]_0_:[polyester]_0_ 1:10.

^d)^ *k*_obs_ determined from the linear fit of the Ln[(weight)_t_/(weight)_0_] versus time plot between 95% and 80% weight. Depolymerizations performed under N_2_ flow rate 25 mL min^−1^, 230 °C, [catalyst]_0_:[polyester]_0_ 1:10.

^e)^ Polyester conversion corresponds to the total weight loss of polyester after 10 h. Depolymerizations performed under N_2_ flow rate 25 mL min^−1^, 230 °C, [catalyst]_0_:[polyester]_0_ 1:10.

^f)^ Selectivity determined by GCMS.

^g)^ Depolymerization product identified by NMR, GC‐MS, and X‐ray crystallography. See Supporting Information for further information. Depolymerizations performed using Kugelrohr distillation apparatus, 10 mbar pressure, 230 °C, [catalyst]_0_:[polyester]_0_ 1:10

In summary, an efficient, catalyzed chemical recycling of (epoxide/anhydride) ROCOP‐derived polyesters produces 16‐ or 18‐membered macrolactones. Using a commercial Sn(II) catalyst, the polyester recycling occurred neat in the melt and at temperatures of ∼200 °C to selectively produce the tetralactones. The macrolactone was repolymerized to form an aliphatic polyester that was same as the starting material, demonstrating the potential for this recycling method to allow for multiple closed loop recycles in future. The catalyzed chemical recycling is generalizable to other aliphatic ROCOP‐derived polyesters and provides a useful future opportunity to address sustainability motivated by reducing plastics wastes and accessing low‐energy, high selectivity recycling.

## Supporting Information

The Supporting Information, with experimental methods, characterization, and polymer/monomer purity evaluation data is available free of charge on the ACS Publications website (PDF).

The authors have cited additional references within the Supporting Information.^[^ ^43^, ^44^, ^45^, ^46^, ^47^, ^48^, ^49^, ^50^, ^51^ ^]^

## Conflict of Interests

The authors declare no conflict of interest.

## Supporting information



Supporting Information

## Data Availability

The data that support the findings of this study are available from the corresponding author upon reasonable request.
