# Supplementary material for: From Polymers to Rings and Back Again: Chemical Recycling of Polyesters to Macrolactones
Source: Angew Chem Int Ed Engl. 2025 Mar 27;64(21):e202423478. doi: 10.1002/anie.202423478 (PMC12087820; doi:10.1002/anie.202423478)
Supplement: Supplementary file 1 — Supporting Information [file ANIE-64-e202423478-s001.docx]

**Supporting Information**

**From Polymers to Rings and Back Again:**

**Chemical Recycling of Polyesters to Macrolactones**

Madeleine L. Smith^a^, Thomas M. McGuire,^a^ Ryan W. F. Kerr^a^, and Charlotte K. Williams^a^*

^a^Department of Chemistry, Chemistry Research Laboratory, University of Oxford, 12 Mansfield Rd, Oxford, OX1 3TA, U.K

Charlotte K. Williams: Email: [charlotte.williams@chem.ox.ac.uk](mailto:charlotte.williams@chem.ox.ac.uk)

**Contents**

[1. Materials and Methods 6](#_Toc190444659)

[2. Synthesis of Poly(PO-*alt*-GA) 7](#_Toc190444660)

[3. Synthesis of Poly(PO-*alt*-SA) 7](#_Toc190444661)

[4. Synthesis of Poly(BO-*alt*-GA) 8](#_Toc190444662)

[5. Catalyst testing in ROCOP Recycling: Representative Example Using Sn(II)Oct_2_ as catalyst 8](#_Toc190444663)

[6. Determination of Activation Energy for the Depolymerization of poly(PO-*alt*-GA) 9](#_Toc190444664)

[7. Isothermal Depolymerization of Polyesters by Sn(II)Oct_2_ 9](#_Toc190444665)

[8. Lab-Scale Polyester Recycling by Sn(II)Oct_2_ 10](#_Toc190444666)

[9. Acetyl End-Capped Poly(PO-*alt*-GA) 12](#_Toc190444667)

[10. Polymerization of the Tetralactones: Representative Example with (PO-*alt*-GA) Tetralactone 12](#_Toc190444668)

[11. Computational Details 12](#_Toc190444669)

[12. Additional Information 14](#_Toc190444670)

[Figure S3. ^1^H NMR spectrum (CDCl_3_) of poly(PO-*alt*-GA). 14](#_Toc190444671)

[Figure S4. ^13^C NMR spectrum (CDCl_3_) of poly(PO-*alt*-GA) with expanded view of peaks at δ = 172 ppm corresponding to the carbonyl resonances . 14](#_Toc190444672)

[Figure S5. ^31^P{^1^H} NMR spectrum (CDCl_3_) of poly(PO-*alt*-GA) end group test.. 15](#_Toc190444673)

[Figure S6. SEC trace of poly(PO-*alt*-GA) in THF using polystyrene calibrants. 15](#_Toc190444674)

[Figure S7. ^1^H NMR spectrum (CDCl_3_) of poly(PO-*alt*-SA). 16](#_Toc190444675)

[Figure S8. ^13^C NMR spectrum (CDCl_3_) of poly(PO-*alt*-SA). 16](#_Toc190444676)

[Figure S9. SEC trace of poly(PO-*alt*-SA) in THF against polystyrene calibrants. 17](#_Toc190444677)

[Figure S10. ^1^H NMR spectrum (CDCl_3_) of poly(BO-*alt*-GA). 17](#_Toc190444678)

[Figure S11. ^13^C NMR spectrum (CDCl_3_) of poly(BO-*alt*-GA). 18](#_Toc190444679)

[Figure S12. SEC trace of poly(BO-*alt*-GA) in THF using polystyrene calibrants. 18](#_Toc190444680)

[Figure S13. Weight loss % at 300 °C for each of the catalysts.. 19](#_Toc190444681)

[Table S1. Calculated activation energies for the recycling of poly(PO-*alt*-GA) 19](#_Toc190444682)

[Table S2: Reaction conditions and conversions for lab-scale recycling of poly(PO-*alt*-GA) using Sn(II)Oct_2_ catalyst 20](#_Toc190444683)

[Figure S14. ^13^C NMR spectrum (CDCl_3_) of the reaction product in the lab-scale recycling of poly(PO-*alt*-GA) catalysed by Sn(II)Oct_2_. Depolymerization performed at 230 °C, [Sn(II)Oct_2_]_0_:[poly(PO-*alt*-GA)]_0_ 1:10, 5 mbar pressure. 21](#_Toc190444684)

[Figure S15. ^1^H COSY NMR spectrum (CDCl_3_) of the reaction product in the lab-scale recycling of poly(PO-*alt*-GA) catalysed by Sn(II)Oct_2_. Depolymerization performed at 230 °C, [Sn(II)Oct_2_]_0_:[poly(PO-*alt*-GA)]_0_ 1:10, 5 mbar pressure. 22](#_Toc190444685)

[Figure S16. ^1^H-^13^C HSQC NMR spectrum (CDCl_3_) of the reaction product in the lab-scale recycling of poly(PO-*alt*-GA) catalysed by Sn(II)Oct_2_. Depolymerization performed at 230 °C, [Sn(II)Oct_2_]_0_:[poly(PO-*alt*-GA)]_0_ 1:10, 5 mbar pressure. 23](#_Toc190444686)

[Figure S17. ^1^H-^13^C HMBC NMR spectrum (CDCl_3_) of the reaction product in the lab-scale recycling of poly(PO-*alt*-GA) catalysed by Sn(II)Oct_2_. Depolymerization performed at 230 °C, [Sn(II)Oct_2_]_0_:[poly(PO-*alt*-GA)]_0_ 1:10, 5 mbar pressure. 23](#_Toc190444687)

[Figure S18. Decoupled ^1^H {^1^H} NMR spectrum (CDCl_3_) of the (PO-*alt*-GA) tetralactone at δ 1.24 ppm corresponding to H_d’_ with decoupling from δ 5.26 ppm corresponding to H_c’_. 23](#_Toc190444688)

[Figure S19. Possible isomers of the (PO-*alt*-GA) tetralactone produced from the depolymerization of poly(PO-*alt*-GA). 24](#_Toc190444689)

[Figure S20. GC of the crude product from the lab-scale recycling of poly(PO-*alt*-GA) by Sn(II)Oct_2_. Depolymerization performed at 230 °C, [Sn(II)Oct_2_]_0_:[poly(PO-*alt*-GA)]_0_ 1:10, 5 mbar pressure. 24](#_Toc190444690)

[Figure S21. TGA thermogram of the crude (PO-*alt*-GA) tetralactone from the lab-scale recycling of poly(PO-*alt*-GA) using Sn(II)Oct. N_2_ flow rate 25 mL min^-1^, heating rate 10 °C min^-1^. 25](#_Toc190444691)

[Figure S22. DSC data for the crude (PO-*alt*-GA) tetralactone from the lab-scale recycling of poly(PO-*alt*-GA) by Sn(II)Oct_2_. N_2_ flow rate 25 mL min^-1^, heating rate 10 °C min^-1^. 25](#_Toc190444692)

[Figure S23. Molecular structure determined by single crystal X-ray diffraction of the E *cis* (PO-*alt*-GA) tetralactone isomer produced by poly(PO-*alt*-GA) recycling. Depolymerization performed at 230 °C, [Sn(II)Oct_2_]_0_:[poly(PO-*alt*-GA)]_0_ 1:10, 5 mbar pressure. Structure 001rwfk24. 26](#_Toc190444693)

[Figure S24. Weight *vs*. Time for isothermal recycling of poly(PO-*alt*-GA) at a) 260 °C and b) 290 °C. Depolymerizations conducted under N_2_ flow rate 25 mL min^-1^, [Sn(II)Oct_2_]_0_:[poly(PO-*alt*-GA)]_0_ 1:500. 27](#_Toc190444694)

[Figure S25. Gas-phase FTIR spectra of (PO-*alt*-GA) tetralactone from the lab-scale recycling of poly(PO-*alt*-GA) (top) and for isothermal depolymerization of poly(PO-*alt*-GA) by Sn(II)Oct_2_ at 290 °C, N_2_ flow rate 25 mL min^-1^, [catalyst]_0_:[poly(PO-*alt*-GA)]_0_ 1:10 (bottom). 27](#_Toc190444695)

[Figure S26. Weight *vs*. Time for poly(PO-*alt*-GA) held at 230 °C for 10 h, N_2_ flow rate 25 mL min^-1^. This data shows the polymer is stable without addition of catalyst at 230 °C. 28](#_Toc190444696)

[Figure S27. Weight *vs*. Time for isothermal recycling of poly(PO-*alt*-GA) at 230 °C. All data is fit to an exponential decay and the rate constant for recycling at each catalyst loading is presented. Depolymerizations conducted under N_2_ flow rate 25 mL min^-1^, [Sn(II)Oct_2_]_0_:[poly(PO-*alt*-GA)]_0_ a) 1:10, b) 1:50, c) 1:100 and d) 1:500. 28](#_Toc190444697)

[Figure S28. Ln[weight_t_/weight_0_] *vs*. Time for isothermal recycling of poly(PO-*alt*-GA). All plots shows a linear fit between 5-15 % conversion of polymer. Depolymerizations were conducted under N_2_ flow rate 25 mL min^-1^, [Sn(II)Oct_2_]_0_:[poly(PO-*alt*-GA)]_0_ a) 1:10, b) 1:50, c) 1:100 and d) 1:500. 29](#_Toc190444698)

[Figure S29. a) Weight vs. Time for isothermal recycling of poly(PO-alt-GA) at 230 °C with different catalyst loadings. b) Plot of Ln[*k*_obs_] vs. Ln[cat]. The kobs value was determined from the linear fit of the Ln[weight_t_/weight_0_] vs time plot between 5-15 % conversion. Depolymerizations performed under N_2_ flow rate 25 mL min^-1^, 230 °C, [Sn(II)Oct_2_]_0_:[poly(PO-*alt*-GA)]_0_ 1:10-500. 30](#_Toc190444699)

[Figure S30. ^1^H NMR spectrum (CDCl_3_) for acetyl end-capped poly(PO-*alt*-GA). 30](#_Toc190444700)

[Figure S31. ^31^P{^1^H} NMR spectrum (CDCl_3_) for acetal end-capped poly(PO-*alt*-GA) end group test. No peaks relating to hydroxyl end groups are observed. 31](#_Toc190444701)

[Figure S32. Weight *vs*. Time for isothermal recycling of acetyl and hydroxyl end-capped poly(PO-*alt*-GA) at 230 °C. Depolymerizations conducted under N_2_ flow rate 25 mL min^-1^, [catalyst]_0_:[poly(PO-*alt*-GA)]_0_ 1:100. 31](#_Toc190444702)

[Figure S33. Weight *vs*. Time for isothermal recycling of poly(PO-*alt*-GA) by Sn(O*^n^*Bu)_2_ and Sn(II)(Oct)_2_ at 230 °C. Depolymerizations conducted under N_2_ flow rate 25 mL min^-1^, [catalyst]_0_:[poly(PO-*alt*-GA)]_0_ 1:100. 32](#_Toc190444703)

[Figure S34. Ln[weight_t_/weight_0_] *vs*. Time for isothermal recycling of poly(PO-*alt*-GA) by Sn(II)(O*^n^*Bu)_2_ at 230 °C. The plot shows a linear fit between 5-15 % conversion of polymer. Depolymerizations conducted under N_2_ flow rate 25 mL min^-1^, [catalyst]_0_:[poly(PO-*alt*-GA)]_0_ 1:100. 32](#_Toc190444704)

[Figure S35. Calculated -ΔG° values for the possible cyclic reaction products from the depolymerization of poly(PO-*alt*-GA) relative to propylene oxide and glutaric anhydride. 33](#_Toc190444705)

[Table S3. Calculated -ΔG° values for the possible cyclic reaction products from the depolymerization of poly(PO-*alt*-GA). 33](#_Toc190444706)

[Table S4. Screening of various catalysts for the ROP of the (PO-*alt*-GA) tetralactone.*^[a]^* 36](#_Toc190444708)

[Figure S36. ^1^H NMR spectrum (CDCl_3_) of the recycled poly(PO-*alt*-GA) after 90 h. ROP of (PO-*alt*-GA) tetralactone performed neat at 150 °C, [Sn(II)Oct_2_]_0_:[benzyl alcohol]_0_:[tetralactone]_0_ 1:1:50. 36](#_Toc190444709)

[Figure S37. SEC trace of the recycled poly(PO-*alt*-GA) after 90 h and SEC trace of virgin polymer. ROP of (PO-*alt*-GA) tetralactone performed neat at 150 °C, [Sn(II)Oct_2_]_0_:[benzyl alcohol]_0_:[tetralactone]_0_ 1:1:50. 37](#_Toc190444710)

[Figure S38. Van’t Hoff analysis of the macrolactone polymerization. ROP of (PO-*alt*-GA) tetralactone performed neat at 140 °C, 150 °C and 160 °C, [Sn(II)Oct_2_]_0_:[benzyl alcohol]_0_:[tetralactone]_0_ 1:1:50. [M]_eq_ determined by ^1^H NMR. 37](#_Toc190444711)

[Figure S39. Weight *vs*. Temperature for recycling of poly(PO-*alt*-SA) by Sn(II)Oct_2_. Depolymerizations conducted under N_2_ flow rate 25 mL min^-1^, heating rate 2 °C min^-1^, [Sn(II)Oct_2_]_0_:[poly(PO-*alt*-SA)]_0_ 1:500. 38](#_Toc190444712)

[Figure S40. Weight *vs*. Temperature for recycling of poly(BO-*alt*-GA). Depolymerizations conducted under N_2_ flow rate 25 mL min^-1^, heating rate 2 °C min^-1^, [Sn(II)Oct_2_]_0_:[poly(BO-*alt*-GA)]_0_ 1:500. 38](#_Toc190444713)

[Figure S41. Weight *vs*. Time for poly(PO-*alt*-SA) held at 230 °C for 10 h, N_2_ flow rate 25 mL min^-1^. 38](#_Toc190444714)

[Figure S42. Weight *vs*. Time for poly(BO-*alt*-GA) held at 230 °C for 10 h, N_2_ flow rate 25 mL min^-1^. 39](#_Toc190444715)

[Figure S43. a) Weight *vs*. Time for isothermal recycling of poly(PO-*alt*-SA) at 230 °C. The data is fit to an exponential decay and the rate constant for recycling is shown. b) Ln[weight_t_/weight_0_] *vs*. Time for isothermal recycling of poly(PO-*alt*-SA) by at 230 °C. The plot shows a linear fit between 5-15 % conversion of the polymer. Depolymerization conducted under N_2_ flow rate 25 mL min^-1^, [Sn(II)Oct_2_]_0_:[poly(PO-*alt*-SA)]_0_ 1:10. 39](#_Toc190444716)

[Figure S44. a) Weight *vs*. Time for isothermal recycling of poly(BO-*alt*-GA) at 230 °C. The data is fit to an exponential decay and the rate constant for recycling is shown. b) Ln[weight_t_/weight_0_] *vs*. Time for isothermal recycling of poly(BO-*alt*-GA) at 230 °C. The plot shows a linear fit between 5-15% conversion of the polymer. Depolymerization conducted under N_2_ flow rate 25 mL min^-1^, [catalyst]_0_:[poly(BO-*alt*-GA)]_0_ 1:10. 39](#_Toc190444717)

[Figure S45. ^1^H NMR spectrum (CDCl_3_) of the crude product from the lab-scale recycling of poly(PO-*alt*-SA). Depolymerization performed at 210 °C, 20 mbar [Sn(II)Oct_2_]_0_:[ poly(PO-*alt*-SA)]_0_ 1:10. Splitting of the peaks at d’, c’, b’ and a’ are due to the 4 different regio- and stereo- isomers of the tetralactone. 40](#_Toc190444718)

[Figure S46. ^13^C NMR spectrum (CDCl_3_) of the crude product from the lab-scale recycling of poly(PO-*alt*-SA). Depolymerization performed at 210 °C, 20 mbar [Sn(II)Oct_2_]_0_:[ poly(PO-*alt*-SA)]_0_ 1:10. 41](#_Toc190444719)

[Figure S47. GC of the crude product from the lab-scale recycling of poly(PO-*alt*-SA). Depolymerization performed at 210 °C, 20 mbar [Sn(II)Oct_2_]_0_:[ poly(PO-*alt*-SA)]_0_ 1:10. 41](#_Toc190444720)

[Figure S48. ^1^H NMR spectrum (CDCl_3_) of the crude product from the lab-scale recycling of poly(BO-*alt*-GA). Depolymerization performed at 230 °C, 4 mbar, [Sn(II)Oct_2_]_0_:[ poly(BO-*alt*-GA)]_0_ 1:10. 42](#_Toc190444721)

[Figure S49. ^13^C NMR spectrum (CDCl_3_) of the crude product from the lab-scale recycling of poly(BO-*alt*-GA). Depolymerization performed at 230 °C, 4 mbar [Sn(II)Oct_2_]_0_:[ poly(BO-*alt*-GA)]_0_ 1:10. 42](#_Toc190444722)

[Figure S50. GC of the crude product from the lab-scale recycling of poly(BO-*alt*-GA). Depolymerization performed at 230 °C, 4 mbar [Sn(II)Oct_2_]_0_:[ poly(BO-*alt*-GA)]_0_ 1:10. 43](#_Toc190444723)

[Figure S51. Na^+^ mass spectrum of the (PO-*alt*-SA) tetralactone collected from the lab-scale recycling of poly(PO-*alt*-SA) (top spectrum) and theoretical Na^+^ mass spectrum of the (PO-*alt*-SA) tetralactone (bottom). Depolymerization performed at 210 °C, 20 mbar [Sn(II)Oct_2_]_0_:[ poly(PO-*alt*-SA)]_0_ 1:10. 44](#_Toc190444724)

[Figure S52. Na^+^ mass spectrum of the (BO-*alt*-GA) tetralactone collected from the lab-scale recycling of poly(BO-*alt*-GA) (top spectrum) and theoretical Na^+^ mass spectrum of the (BO-*alt*-GA) tetralactone (bottom). Depolymerization performed at 230 °C, 4 mbar [Sn(II)Oct_2_]_0_:[ poly(BO-*alt*-GA)]_0_ 1:10. 45](#_Toc190444725)

[13. Discussion of Regio- and Stereo- Selectivity 47](#_Toc190444726)

[14. Crystallographic Data 48](#_Toc190444727)

[15. References 49](#_Toc190444729)

## Materials and Methods

All experiments were carried out under N_2_ using standard Schlenk/glovebox techniques unless otherwise stated. Tin(II) 2-ethylhexanoate (Sn(II)(Oct)_2_) was purchased from Sigma Aldrich and used as received. Propylene oxide (PO) and butylene oxide (BO) were purchased from Sigma Aldrich, dried over calcium hydride and purified by fractional distillation, followed by degassing with N_2_ and stored under N_2_. All solvents used were anhydrous, unless otherwise stated. THF and toluene were obtained from an SPS system, degassed by several freeze-pump-thaw cycles and stored over 3 Å molecular sieves, under nitrogen. 4-Methylbenzyl alcohol was purchased from Sigma Aldrich and recrystallised from anhydrous diethyl ether prior to use. P_2_-*^t^*Bu in 2.0 M THF was purchased from Sigma Aldrich and used as received. Amberchrom™ 50WX8 was purchased from Sigma Aldrich and used as received.

Purification of glutaric anhydride (GA, Sigma Aldrich) was achieved by stirring it in dry acetic anhydride under reflux at 80 °C. The acetic anhydride was then removed in vacuo. The resultant white powder was recrystallised from diethyl ether and subsequently sublimed twice under vacuum at 48 °C. Diglycolic anhydride (DGA, Acros Organics) was purified through recrystallisation from dry acetic anhydride, followed by two sublimations at 85 °C. Succinic anhydride (SA, Sigma Aldrich) was purified by stirring it in dry acetic anhydride under reflux at 80 °C. The acetic anhydride was then removed, in vacuo, at 50 °C. The resultant white powder was recrystallised from THF and subsequently sublimed twice, under vacuum, at 40 °C.

Magnesium(II) chloride, zinc(II) chloride (anhydrous), calcium(II) 2-ethylhexanoate and barium(II) 2-ethylhexanoate were purchased from Sigma-Aldrich and used as received. Zirconium(IV) 2-ethylhexanoate and tin (IV) 2-ethylhexanoate diisopropoxide was purchased from Alfa Aesar and used as received. Zinc(II) 2-ethylhexanoate was purchased from Fluorochem and used as received. Bismuth(III) 2-ethylhexanoate was purchased from Thermo Scientific Chemicals and used as received.

**Size exclusion chromatography (SEC)** was carried out on a Shimadzu LC-20AD instrument using two PSS SDV linear M columns in series, with a THF eluent. Measurements were conducted at 30 °C, with a flow rate of 1 mL/min. Samples were detected with a differential refractive index (RI) detector. Number-average molar mass (*M*_n,SEC_),and dispersities, (*Ð*_M_ = *M*_w_/*M*_n_)) were calculated against a series of well-defined polystyrene calibrants with a mass range of 500 to 1,000,000 g mol^-1^. The polymer samples were dissolved in HPLC-grade THF, at a concentration of *ca* 10 mg/mL, and filtered through a 0.2 µm microfilter prior to analysis.

**Differential scanning calorimetry (DSC)** was performed using a TA discovery 25 auto. Experiments were performed under N_2_ flow (50 mL/min) using aluminium TZERO pans. Samples (2–5 mg) were equilibrated at 40 °C then heated at a rate of 10 °C/min to 200 °C, and held at 200 °C, for 2 minutes. The sample was then cooled, at a rate of 10 °C/min, to –80 °C and held at –80 °C for 2 minutes. The sample was then heated, at a rate of 10 °C/min, to 200 °C and cooled, at a rate 10 °C/min, to –80 °C for 3 successive cycles. Thermal data is reported from the second heating cycle.

**Thermal gravimetric analysis (TGA)** were collected on a TGA5500 System (TA Instruments), equipped with the TRIOS software package. Detailed procedures are given in the protocol section.

**NMR** spectra were obtained using a Bruker AVIII HD nanobay NMR spectrometer. Coupling constants are given in Hertz.

**GC-MS** spectra were recorded on an Agilent 7820A equipped with a HP5-MS ultra inert column (30 m length, 0.25 mm internal diameter, 0.25 µm film thickness), a 5977B single quad mass spectrometer, a liquid injection autosampler and He carrier gas. Data was processed using MassHunter software. Tetralactone samples of 5 mg/mL were prepared in dichloromethane with 1µL injected. Samples were loaded on to the column in 1:100 sample:solvent splitter ratio and injection port temperature of 300 °C. The column was pressurised at 9.1 PSI, with a column flow of 1.2 mL/min and total flow of 22.12 ml/min. Following equilibration of the column oven at 40 °C for 1 minute, the temperature was ramped from 40 °C to 300 °C at a rate of 10 °C/min and held at 300 °C for 3 minutes. The MS source and quadrupole temperature was 230 °C and 150 °C, respectively.

**Turnover Frequency (TOF)** calculations were performed using mass loss against time plots from 5-20% mass loss of the polymer over time.

## Synthesis of Poly(PO-*alt*-GA)

Inside a nitrogen filled glovebox, glutaric anhydride (5.00 g, 44 mmol, 200 equiv.), propylene oxide (6.2 mL, 88 mmol, 400 equiv.), benzyl alcohol (182 μL, 1.8 mmol, 8 equiv.) and 2.0 M P_2_-*^t^*Bu in THF (185 μL, 0.37 mmol, 1 equiv.) were added to a dried vial, equipped with a magnetic stirrer bar. The vial was sealed with a melamine-cap containing a Teflon inlay, and further sealed with electrical insulation tape. This sealed vial was then heated to 60 °C, until the polymerization reached completion. The reaction mixture was then exposed to air and the polymer purified by stirring over ~1 g Amberchrom™ 50WX8 overnight. The Amberchrom™ 50WX8 was removed by filtration and the polymer passed through a silica plug to give a clear, viscous gel (3.99 g, 53 % yield).

***M*_n, SEC_** = 4800 g mol^-1^ (1.83)

**T_d, 5%_** = 313 °C

***T*_g_** = -34 °C

**^1^H NMR spectroscopy (600 MHz, CDCl_3_)** δ 5.27 – 5.08 (m, 1H, **H_c_**), 4.23 – 4.13 (m, 1H, **H_e_**), 4.08 - 3.97 (m, 1H, **H_e_**), 2.48 – 2.27 (m, 4H, **H_a_**), 2.03 – 1.82 (m, 2H, **H_b_**), 1.29 – 1.18 (m, 3H, **H_d_**).

**^13^C NMR spectroscopy (151 MHz, CDCl_3_)** δ 172.5 (**C_f_**), 172.1 (**C_f_**), 128.5 (**C_benzyl_**), 128.2 (**C_benzyl_**), 128.1 (**C_benzyl_**), 68.2 (**C_c_**), 65.9 (**C_e_**), 33.3 (**C_a_**), 19.9 (**C_b_**), 16.5 (**C_d_**).

## Synthesis of Poly(PO-*alt*-SA)

Inside a nitrogen filled glovebox, succinic anhydride (2.19 g, 22 mmol, 200 equiv.), propylene oxide (3.1 mL, 44 mmol, 400 equiv.), benzyl alcohol (91 μL, 0.88 mmol, 8 equiv.) and 2.0 M P_2_-*^t^*Bu in THF (55 μL, 0.11 mmol, 1 equiv.) were added to a dried vial, equipped with a magnetic stirrer bar. The vial was sealed with a melamine-cap containing a Teflon inlay, and further sealed with electrical insulation tape. This sealed vial was then heated to 60 °C until the polymerization reached completion. The reaction mixture was then exposed to air and the polymer purified by precipitation in methanol followed by a silica plug to give a viscous gel (1.85 g, 53 % yield).

***M*_n, SEC_** = 5300 g mol^-1^ (2.22)

**T_d, 5%_** = 294 °C

***T*_g_** = -11 °C

**^1^H NMR spectroscopy (400 MHz, CDCl_3_)** δ 5.17 – 5.10 (m, 1H, **H_c_**), 4.22 – 4.01 (m, 2H, **H_b_**), 2.72 – 2.57 (m, 4H, **H_a_**), 1.28 – 1.13 (m, 3H, **H_d_**).

**^13^C NMR spectroscopy (101 MHz, CDCl_3_) δ** 172.1 (**C_e_**), 171.7 (**C_e_**), 128.7 (**C_benzyl_**), 128.4 (**C_benzyl_**), 68.7 (**C_c_**), 66.3 (**C_b_**), 29.1 (**C_a_**), 16.5 (**C_d_**).

## Synthesis of Poly(BO-*alt*-GA)

Inside a nitrogen filled glovebox, glutaric anhydride (1.13 g, 9.9 mmol, 200 equiv.), butylene oxide (1.7 mL, 20 mmol, 400 equiv.), benzyl alcohol (41 μL, 0.40 mmol, 8 equiv.) and 2.0 M P_2_-*^t^*Bu in THF (25 μL, 0.050 mmol, 1 equiv.) were added to a dried vial, equipped with a magnetic stirrer bar. The vial was sealed with a melamine-cap containing a Teflon inlay, and further sealed with electrical insulation tape. This sealed vial was then heated to 60 °C until the polymerization reached completion. The reaction mixture was then exposed to air and the polymer purified by precipitation in methanol followed by a silica plug to give a clear, viscous gel (0.74 g, 40 % yield).

***M*_n, SEC_** = 3000 g mol^-1^ (2.29)

**T_d, 5%_** = 314 °C

***T*_g_** = -43.3 °C

**^1^H NMR spectroscopy (400 MHz, CDCl_3_) δ** 5.08 – 4.95 (m, 1H, **H_c_**), 4.24 (ddt, *J* = 12.0, 4.2, 2.1 Hz, 1H, **H_e_**), 4.08 – 3.97 (m, 1H, **H_e_**), 2.38 (m, *J* = 7.4, 2.1 Hz, 4H, **H_a_**), 1.93 (m, *J* = 13.0, 7.1 Hz, 2H, **H_b_**), 1.69 – 1.47 (m, 2H, **H_d_**), 1.02 – 0.86 (m, 3H, **H_f_**).

**^13^C NMR spectroscopy** (101 MHz, CDCl_3_) δ 172.7 (**C_g_**), 172.6 (**C_g_**), 128.7 (**C_benzyl_**), 128.3 (**C_benzyl_**), 72.9 (**C_c_**), 64.8 (**C_e_**), 33.4 (**C_a_**), 33.3 (**C_a_**), 33.1 (**C_a_**), 33.1 (**C_a_**), 24.0 (**C_b_**), 20.2 (**C_d_**), 9.6 (**C_f_**).

## Catalyst testing in ROCOP Recycling: Representative Example Using Sn(II)Oct_2_ as catalyst

In the glovebox, stock solutions of poly(PO-*alt*-GA) (1.00 M, 344 mg of poly(PO-*alt*-GA) in 2.0 mL THF) and Sn(II)Oct_2_ (0.01 M, 12.2 mg of Sn(II)Oct_2_ in 3.0 mL THF) were prepared. The poly(PO-*alt*-GA) stock solution (100 µL, 0.1 mmol, 500.0 equiv.) was added to a vial containing Sn(II)(Oct)_2_ (20.0 µL, 2.00 x 10 ^–4^ mmol, 1.0 equiv.). The Sn(II)(Oct)_2_- poly(PO-*alt*-GA) solution was thoroughly mixed before being transferred to an aluminium TGA crucible. The crucible was placed under vacuum, for 30 minutes, to remove the solvent, before being crimped in the glovebox with a hermetic seal. The crucible was then transferred to a TGA instrument for solid-state recycling testing using the method outlined below.

1. N­_2_ flow of 25.0 mL min^-1^
2. Equilibrate at 30 °C
3. Increase temperature to 100 °C
4. Heat at 2 °C min^-1^ up to 400 °C
5. Heat at 10 °C min^-1^ to 500 °C

The following catalysts were also tested using the same procedure: Ca(II)Oct_2_, Ba(II)Oct_2_, Zn(II)Oct_2_, Zr(IV)Oct_4_, Bi(III)Oct_3_, Sn(IV)Oct_2_, Mg(II)Cl_2_ and Zn(II)Cl_2_. A 0.01 M stock of each catalyst was prepared by dissolving the catalyst in THF in the amounts given below.

The sample mass at 100 °C was used to normalise the data (and account for any minor solvent losses which occur below this temperature).

| Catalyst | Stock Solution Concentration (M) | Volume THF (mL) | Mass (mg) | Recycling onset temperature (°C)*^[a]^* | Weight loss % at 300 °C |
| --- | --- | --- | --- | --- | --- |
| Ca(II)Oct_2_ | 0.01 | 3 | 9.8 | 291 | 5.8 |
| Ba(II)Oct_2_ | 0.01 | 3 | 12.7 | 303 | 4.5 |
| Zn(II)Oct_2_ | 0.01 | 3 | 10.6 | 299 | 5.1 |
| Zr(IV)Oct_2_ | 0.01 | 3 | 19.9 | 277 | 10.2 |
| Bi(III)Oct_2_ | 0.01 | 3 | 19.2 | 282 | 6.6 |
| Sn(II)Oct_2_ | 0.01 | 3 | 12.2 | 216 | 60.6 |
| Sn(IV)Oct_2_ | 0.01 | 3 | 14.9 | 290 | 7.0 |
| Mg(II)Cl_2_ | 0.01 | 10 | 9.5 | 282 | 8.5 |
| Zn(II)Cl_2_ | 0.01 | 10 | 13.6 | 290 | 7.6 |

*^[a]^*Recycling onset temperature = temperature at which 5% weight loss occurs.

## Determination of Activation Energy for the Depolymerization of poly(PO-*alt*-GA)

In the glovebox, stock solutions of poly(PO-*alt*-GA) (1.00 M, 344 mg of poly(PO-*alt*-GA) in 2.00 mL THF) and Sn(II)Oct_2_ (0.01 M, 12.2 mg of Sn(II)Oct_2_ in 3.00 mL THF) were prepared. The poly(PO-*alt*-GA) stock solution (100 µL, 0.1 mmol, 500.0 equiv.) was added to a vial containing Sn(II)(Oct)_2_ (20.0 µL, 2.00 x 10 ^–4^ mmol, 1.0 equiv.). The Sn(II)(Oct)_2_- poly(PO-*alt*-GA) solution was thoroughly mixed before being transferred to an aluminium TGA crucible. The crucible was placed under vacuum, for 30 minutes, to remove the solvent, before being crimped in the glovebox with a hermetic seal. The crucible was then transferred to a TGA instrument for solid-state depolymerization using the method outlined below.

1. N­_2_ flow of 25.0 mL min^-1^
2. Equilibrate at 30 °C
3. Increase temperature to 100 °C
4. Heat at specified heating rate (see below) up to 400 °C
5. Heat at 10 °C min^-1^ to 600 °C

The experiment was performed using 3 heating rates of 3, 6 and 10 °C min^-1^ (step 4.) with catalyst. Using the same heating rates, the experiments were repeated without catalyst. Flynn-Wall analysis was then applied to both sets of data using TRIOS software. In Flynn-Wall analysis, the temperature, T, at which a specified conversion is reached, is measured for different heating rates, β. The activation energy is then determined from the gradient of the plot of logβ against 1/T.^37^ The activation energies were determined for 15, 17,19, 21, 23 and 25 % conversions both with, and without, the Sn(II)Oct_2_ catalyst and the average of these values compared. See Table S1 for results.

## Isothermal Depolymerization of Polyesters by Sn(II)Oct_2_

In the glovebox, stock solutions of poly(PO-*alt*-GA) (1.00 M, 344 mg of poly(PO-*alt*-GA) in 2.00 mL THF) and Sn(II)Oct_2_ (0.01 M, 12.2 mg of Sn(II)Oct_2_ in 3.00 mL THF) were prepared. The poly(PO-*alt*-GA) stock solution (100 µL, 0.1 mmol, 500.0 equiv.) was added to a vial containing Sn(II)(Oct)_2_ (20.0 µL, 2.00 x 10 ^–4^ mmol, 1.0 equiv.). The Sn(II)(Oct)_2_- poly(PO-*alt*-GA) solution was thoroughly mixed before being transferred to an aluminium TGA crucible. The crucible was placed under vacuum, for 30 minutes, to remove the solvent, before being crimped in the glovebox with a hermetic seal. The crucible was then transferred to a TGA instrument for solid-state depolymerization using the method outlined below.

1. N­_2_ flow of 25.0 mL min^-1^
2. Equilibrate at 30 °C
3. Isotherm at 230 °C for 600 minutes
4. Ramp 10 °C min^-1^ to 600 °C

The catalyst loading and polyester were varied as required. High catalyst loadings were used to minimize the length of time required for the depolymerization reaction. 230 °C was selected as the optimum depolymerization temperature; at higher temperatures, thermolysis of the polyester resulted in a loss of selectivity (Figures S24 and S25) whilst at lower temperatures, the depolymerization reaction is limited by the low volatility of the produced tetralactone. No decomposition of poly(PO-*alt*-GA) was observed at 230 °C in the absence of catalyst.

To account for residual solvent loss from the polymer films, which will also be detected as a mass loss in TGA, data from the first 2.0 minutes of the run was removed from the analysis. The mass at 2.0 minutes was then taken as the polymer/catalyst initial mass and used to calculate the change in weight % of the sample.

**Figure S1. Weight *vs.* Time for a representative isothermal depolymerization. The initial 2 min of the depolymerization shows weight loss due to loss of residual solvent.**

The residual mass of the Sn was accounted for as follows: 1) Take the mass at time = 2.0 minutes as the initial polymer and catalyst mass. 2) Subtract the theoretical %mass of Sn in the run: e.g. for a loading of 1:10 Sn(II)(Oct)_2_: poly(PO-*alt*-GA): % mass of Sn = (Mr of Sn)/[(Mr of Sn(II)Oct_2_) + 10(Mr of poly(PO-*alt*-GA) repeat unit (172.20))] = (118.71)/[(405.12)+ 10(172)] = 5.58%. Therefore, mass of initial polymer/catalyst film = 94.42% of mass at 2.0 minutes. 3) Take the mass in step 2) as the initial mass for calculation of the change in mass% of the sample.

## Lab-Scale Polyester Recycling by Sn(II)Oct_2_

In the glovebox, a stock solution of Sn(II)Oct_2_ (1.00 M, 0.20 g of Sn(II)Oct_2_ in 0.50 mL THF) was prepared. The Sn(II)Oct_2_ stock solution (21 μL, 0.02 mmol, 1 equiv.) was added to a pre-weighed 50 mL round bottom flask containing poly(PO-*alt*-GA) (0.37 g, 2.07 mmol, 100 equiv.). A pre-weighed collection flask was added and the flasks connected to the Kugelrohr distillation apparatus. The flasks were placed under reduced pressure (10 mbar) and heated to 230 °C, with a rotation speed of 40 rpm. At the end of the reaction, atmospheric pressure was re-established in the system and the flasks were disconnected from the apparatus. The mass balance was calculated and the flask contents analysed by NMR spectroscopy.

The residual mass of the Sn was accounted for by subtracting the theoretical mass of Sn from the final mass of the starting round bottom flask.

For all runs, >99% mass was conserved during the reaction.

The catalyst loading, polyester, reaction temperature, reaction pressure and reaction time were varied as required. See Table S2 for further details.


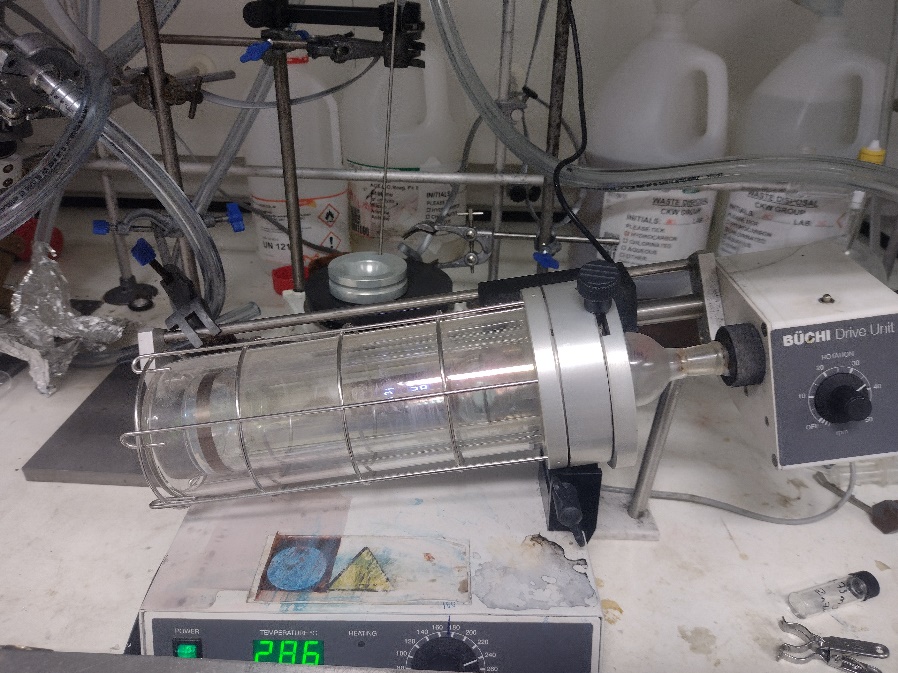


**Figure S2. Image of the Kugelrohr apparatus used in the lab-scale polyester recycling by Sn(II)Oct_2_.**

**(PO-*alt*-GA)** **Tetralactone from recycling of poly(PO-*alt*-GA):**

**^1^H NMR spectroscopy (400 MHz, CDCl_3_) δ** 5.19 (m, *J* = 10.0, 6.1 Hz, 2H, **H_c’_**), 4.38 - 4.21 (m, 2H, **H_e’_**), 4.06 – 3.87 (m, 2H, **H_e’_**), 2.40 (m, *J* = 14.0, 9.9, 5.7 Hz, 8H, **H_a’_**), 2.11 – 1.88 (m, 4H, **H_b’_**), 1.29 – 1.21 (m, 6H, **H_d’_**). See Figure 2.

**^13^C NMR spectroscopy (151 MHz, CDCl_3_) δ** 172.7 (**C_f’_**), 172.6 (**C_f’_**), 172.6 (**C_f’_**), 172.6 (**C_f’_**), 172.5 (**C_f’_**), 172.5 (**C_f’_**), 172.4 (**C_f’_**), 172.3 (**C_f’_**), 68.3 (**C_c’_**), 68.4 (**C_c’_**), 68.3 (**C_c’_**), 68.3 (**C_c’_**), 66.4 (**C_e’_**), 66.3 (**C_e’_**), 66.2 (**C_e’_**), 66.1 (**C_e’_**), 33.8 (**C_a’_**), 33.6 (**C_a’_**), 33.6 (**C_a’_**), 33.4 (**C_a’_**), 33.4 (**C_a’_**), 33.1 (**C_a’_**), 33.1 (**C_a’_**), 32.7 (**C_a’_**), 20.8 (**C_b’_**), 20.6 (**C_b’_**), 20.5 (**C_b’_**), 20.4 (**C_b’_**), 16.5 (**C_d’_**), 16.4 (**C_d’_**), 16.4 (**C_d’_**). See Figure S13.

***m/z* = 344.11 (*m/z*_theo_ = 344.15).**

See Figures 3 and S23 for molecular structure determined by single crystal X-ray diffraction.

**(PO-*alt*-SA) Tetralactone from recycling of poly(PO-*alt*-SA):**

**^1^H NMR spectroscopy (600 MHz, CDCl_3_) δ** 5.26 – 5.08 (m, 2H, **H_c’_**), 4.48 – 3.75 (m, 4H, **H_b’_**), 2.82 – 2.41 (m, 8H, **H_a’_**), 1.32 – 1.10 (m, 6H, **H_d’_**). See Figure S43.

**^13^C NMR spectroscopy (101 MHz, CDCl_3_)** δ 171.6 (**C_e’_**), 171.3 (**C_e’_**), 171.3 (**C_e’_**), 170.9 (**C_e’_**), 68.9 (**C_c’_**), 68.4 (**C_c’_**), 68.4 (**C_c’_**), 68.1 (**C_c’_**), 66.3 (**C_b’_**), 66.2 (**C_b’_**), 66.1 (**C_b’_**), 29.2 (**C_a’_**), 29.1 (**C_a’_**), 29.1 (**C_a’_**), 29.1 (**C_a’_**), 29.0 (**C_a’_**), 29.0 (**C_a’_**), 16.1 (**C_d’_**), 16.0 (**C_d’_**), 15.9 (**C_d’_**), 15.9 (**C_d’_**). See Figure S44.

***m/z* = 316.29 (*m/z*_theo_ = 316.12).**

**(BO-*alt*-GA) Tetralactone from recycling of poly(BO-*alt*-GA):**

**^1^H NMR spectroscopy (400 MHz, CDCl_3_) δ** 5.16 – 5.00 (m, 2H, **H_c’_**), 4.44 – 3.79 (m, 4H, **H_e’_**), 2.58 –2.40 (m, 8H, **H_a’_**), 2.19 –1.85 (m, 4H, **H_b’_**), 1.75 – 1.39 (m, 4H, **H_d’_**), 0.93 (m, 6H, **H_f’_**). See Figure S46.

**^13^C NMR spectroscopy (101 MHz, CDCl_3_) δ** 172.7 (**C_g’_**), 72.8 (**C_c’_**), 72.8 (**C_c’_**), 72.6 (**C_c’_**), 65.3 (**C_e’_**), 65.1 (**C_e’_**), 64.9 (**C_e’_**), 64.8 (**C_e’_**), 33.7 (**C_a’_**), 33.5 (**C_a’_**), 33.4 (**C_a’_**), 33.1 (**C_a’_**), 24.0 (**C_b’_**), 24.0 (**C_b’_**), 23.9 (**C_b’_**), 20.5 (**C_d’_**), 20.5 (**C_d’_**), 9.7 (**C_f’_**), 9.6 (**C_f’_**). See Figure S47.

***m/z* = 372.17 (*m/z*_theo_ = 372.18).**

See Figure S52 for molecular structure determined by single crystal X-ray diffraction.

## Acetyl End-Capped Poly(PO-*alt*-GA)

The acetyl end-capped polymer was prepared according to the literature.^43^

**^1^H NMR spectroscopy (400 MHz, CDCl_3_) δ** 5.18 – 5.06 (m, 1H, **H_c_**), 4.17 (ddt, *J* = 11.8, 3.6, 1.5 Hz, 1H, **H_e_**), 4.04 (m, *J* = 11.8, 4.3 Hz, 1H, **H_e_**), 2.37 (m, *J* = 10.0, 7.3 Hz, 4H, **H_a_**), 2.01 – 1.85 (m, 2H), 1.26 – 1.17 (m, 3H, **H_d_**).

## Polymerization of the Tetralactones: Representative Example with (PO-*alt*-GA) Tetralactone

In the glovebox, a stock solution of the (PO-*alt*-GA) tetralactone (1.00 M, 84 mg of (PO-*alt*-GA) tetralactone in 0.24 mL THF), benzyl alcohol (0.1 M, 20.7 μL of benzyl alcohol in 2.00 mL THF) and Sn(II)Oct_2_ (0.1 M, 40.5 mg of Sn(II)Oct_2_ in 1.00 mL THF) was prepared. The (PO-*alt*-GA) tetralactone stock solution (200 μL, 0.2 mmol, 50 equiv.) and Sn(II)Oct_2_ stock solution (40 μL, 0.004 mmol, 1 equiv.) was added to a vial containing benzyl alcohol stock solution (40 μL, 0.004 mmol, 1 equiv.). The Sn(II)(Oct)_2_-(PO-*alt*-GA) tetralactone solution was thoroughly mixed before being transferred (60 μL) to an aluminium Tzero DSC crucible. The crucible was placed under vacuum, for 30 minutes, to remove the solvent, before being crimped in the glovebox with a hermetic seal. The crucible was then removed from the glove box and placed in a heating block and heated at 150 °C for 20 h. The crucible was then removed from the heating block and pierced. The content of the crucible was dissolved in CDCl_3_ and analysed by NMR spectroscopy and SEC.

The catalyst, catalyst loading and reaction time were varied as required; see Table S4 for details.

The following catalysts were also tested using the same procedure: P_4_-*^t^*Bu, Y(III)Oct_3_, Ti(O*^i^*Pr)_4_, and Y{N(Si(CH_3_)_3_)_2_}_3_. The Sn(II)Oct_2_ displayed the highest activity of the catalysts tested so was selected for further study.

## Computational Details

Density Functional theory (DFT) calculations were performed using Gaussian16 suite of codes (revision C.01).^44^ Geometries were fully optimised without any symmetry or geometry constraints. Free enthalpies were calculated at 298.15 K within the harmonic approximation for vibrational frequencies. Geometry optimisations were carried out using rwb97xd functional.^45, 46^ The 6-311+g(d,p) basis set was used for the C, H and O atoms. Full coordinates for all structures, together with computed energies and vibrational frequency data, are available via the corresponding Gaussian 16 output files, stored in the open-access digital repository: <https://figshare.com/s/f65f52ec3cd5c17a628e>

See Figure S35 and Table S3 for computational results.

## Additional Information

### Figure S3. ^1^H NMR spectrum (CDCl_3_) of poly(PO-*alt*-GA).

### Figure S4. ^13^C{^1^H} NMR spectrum (CDCl_3_) of poly(PO-*alt*-GA) with expanded view of peaks at δ = 172 ppm corresponding to the carbonyl resonances .

### Figure S5. ^31^P{^1^H} NMR spectrum (CDCl_3_) of poly(PO-*alt*-GA) end group test. Peaks at δ = 146 ppm and δ = 148 ppm correspond to primary and secondary hydroxyl end groups, respectively. Both form as the polymerization is regiorandom.

Figure S6. SEC trace of poly(PO-*alt*-GA) in THF using polystyrene calibrants.

### Figure S7. ^1^H NMR spectrum (CDCl_3_) of poly(PO-*alt*-SA).

### Figure S8. ^13^C NMR spectrum (CDCl_3_) of poly(PO-*alt*-SA).

Figure S9. SEC trace of poly(PO-*alt*-SA) in THF against polystyrene calibrants. Bimodality is observed due to residual protic impurities in the PO and SA monomers.

### Figure S10. ^1^H NMR spectrum (CDCl_3_) of poly(BO-*alt*-GA).

### Figure S11. ^13^C NMR spectrum (CDCl_3_) of poly(BO-*alt*-GA).

Figure S12. SEC trace of poly(BO-*alt*-GA) in THF using polystyrene calibrants.

### Figure S13. Weight loss % at 300 °C for each of the catalysts. Reactions conducted under N_2_ flow rate 25 mL min^-1^, heating rate of 2 °C min^-1^, [catalyst]_0_:[poly(PO-*alt*-GA)]_0_ 1:500.

### Table S1. Calculated activation energies for the recycling of poly(PO-*alt*-GA).*^[a]^*

| **Conversion %** | **Activation energy of depolymerization**  **KJ mol^-1^** | |
| --- | --- | --- |
|  | **[poly(PO-*alt*-GA)]** | **[Sn(II)Oct_2_]:[poly(PO-*alt*-GA)] 1:500** |
| 5% | 284 | 55 |
| 10% | 272 | 73 |
| 15% | 265 | 93 |
| 17% | 262 | 101 |
| 19% | 260 | 107 |
| 21% | 259 | 113 |
| 23% | 258 | 119 |
| 25% | 258 | 124 |
| **Average** | **260** | **110** |

*^[a]^*The activation energy of depolymerization was calculated by Flynn-Wall analysis using TRIOS software. In Flynn-Wall analysis, the temperature, T, at which a specified conversion is reached, is measured for different heating rates, β. Heating rates of 3, 6 and 10 °C min^-1^ were applied to the polymer, both with and without the Sn(II)Oct_2_ catalyst at a loading of [Sn(II)Oct_2_]_0_:[polyester]_0_ 1:500. The activation energy was then determined from the gradient of the plot of logβ against 1/T.^1^ The activation energies were determined for 15, 17,19, 21, 23 and 25 % conversion both with and without the Sn(II)Oct_2_ catalyst and the average of these values compared.

### Table S2: Reaction conditions and conversions for lab-scale recycling of poly(PO-*alt*-GA) using Sn(II)Oct_2_ catalyst

| Polyester | Temperature (°C) | [Sn(II)Oct_2_]_0_:[polyester]_0_ | Vacuum (mbar) | Time (h) | Starting mass of polymer (g) | Residual mass of polymer (g) | Mass of collected depolymerization products (g) | Lactone selectivity (%)*^[a]^* | Conversion to tetralactone (%)*^[b]^* |
| --- | --- | --- | --- | --- | --- | --- | --- | --- | --- |
| Poly(PO-*alt*-GA) | 230 | 1:100 | 10 | 96 | 0.356 | 0.129 | 0.227 | >99 | 64 |
| Poly(PO-*alt*-GA) | 230 | 1:100 | 5 | 24 | 0.318 | 0.166 | 0.150 | >99 | 47 |
| Poly(PO-*alt*-GA) | 230 | 1:10 | 4 | 48 | 0.419 | 0.0871 | 0.3319 | >99 | 79 |
| Poly(PO-*alt*-SA) | 210 | 1:10 | 20 | 24 | 0.290 | 0.105 | 0.180 | >99 | 62 |
| Poly(BO-*alt*-GA) | 230 | 1:10 | 5 | 6 | 0.190 | 0.0819 | 0.108 | 74 | 42 |

*^[a]^*Lactone selectivity calculated by GC-MS. *^[b]^*Conversion calculated from mass balance.

### Figure S14. ^13^C NMR spectrum (CDCl_3_) of the reaction product in the lab-scale recycling of poly(PO-*alt*-GA) catalysed by Sn(II)Oct_2_. Depolymerization performed at 230 °C, [Sn(II)Oct_2_]_0_:[poly(PO-*alt*-GA)]_0_ 1:10, 5 mbar pressure.

### Figure S15. ^1^H COSY NMR spectrum (CDCl_3_) of the reaction product in the lab-scale recycling of poly(PO-*alt*-GA) catalysed by Sn(II)Oct_2_. Depolymerization performed at 230 °C, [Sn(II)Oct_2_]_0_:[poly(PO-*alt*-GA)]_0_ 1:10, 5 mbar pressure.

### Figure S16. ^1^H-^13^C HSQC NMR spectrum (CDCl_3_) of the reaction product in the lab-scale recycling of poly(PO-*alt*-GA) catalysed by Sn(II)Oct_2_. Depolymerization performed at 230 °C, [Sn(II)Oct_2_]_0_:[poly(PO-*alt*-GA)]_0_ 1:10, 5 mbar pressure.

### Figure S17. ^1^H-^13^C HMBC NMR spectrum (CDCl_3_) of the reaction product in the lab-scale recycling of poly(PO-*alt*-GA) catalysed by Sn(II)Oct_2_. Depolymerization performed at 230 °C, [Sn(II)Oct_2_]_0_:[poly(PO-*alt*-GA)]_0_ 1:10, 5 mbar pressure.

### Figure S18. Decoupled ^1^H{^1^H} NMR spectrum (CDCl_3_) of the (PO-*alt*-GA) tetralactone at δ 1.24 ppm corresponding to H_d’_ with decoupling from δ 5.26 ppm corresponding to H_c’_. 4 peaks are observed at δ 1.26, 1.24, 1.24 and 1.23 ppm corresponding to the different possible isomers. These structures are not assigned but the presence of all four confirms that the lactone formation is both stereo- and regiorandom. This is consistent with the polymer structure which is equivalently stereo- and regiorandom.

### Figure S19. Possible isomers of the (PO-*alt*-GA) tetralactone produced from the depolymerization of poly(PO-*alt*-GA).

### Figure S20. GC of the crude product from the lab-scale recycling of poly(PO-*alt*-GA) by Sn(II)Oct_2_. Peaks at 22.45 and 22.51 min correspond to *m/z* = 344.11. This is a match to the theoretical mass of the proposed 18-membered ring (*m/z*_theo_ = 344.15). No other peaks in the GC were observed; selectivity for the tetralactone >99%. Depolymerization performed at 230 °C, [Sn(II)Oct_2_]_0_:[poly(PO-*alt*-GA)]_0_ 1:10, 5 mbar pressure.

####

### Figure S21. TGA thermogram of the crude (PO-*alt*-GA) tetralactone from the lab-scale recycling of poly(PO-*alt*-GA) using Sn(II)Oct. N_2_ flow rate 25 mL min^-1^, heating rate 10 °C min^-1^.

### Figure S22. DSC data for the crude (PO-*alt*-GA) tetralactone from the lab-scale recycling of poly(PO-*alt*-GA) by Sn(II)Oct_2_. N_2_ flow rate 25 mL min^-1^, heating rate 10 °C min^-1^. Melt transitions at 74 and 93 °C are assigned to two of the possible four diastereoisomers, most likely the *cis* and *trans* isomers.


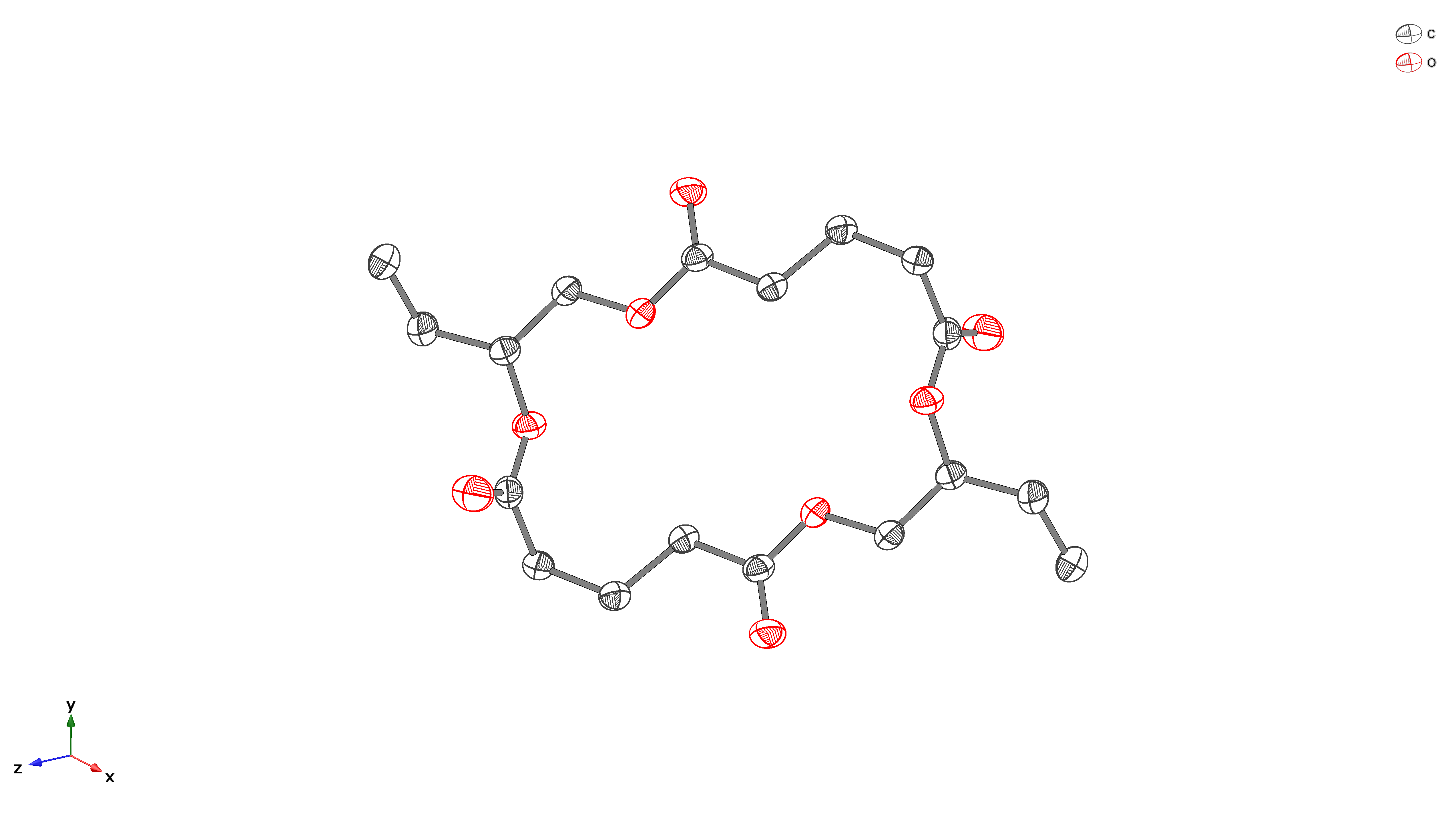


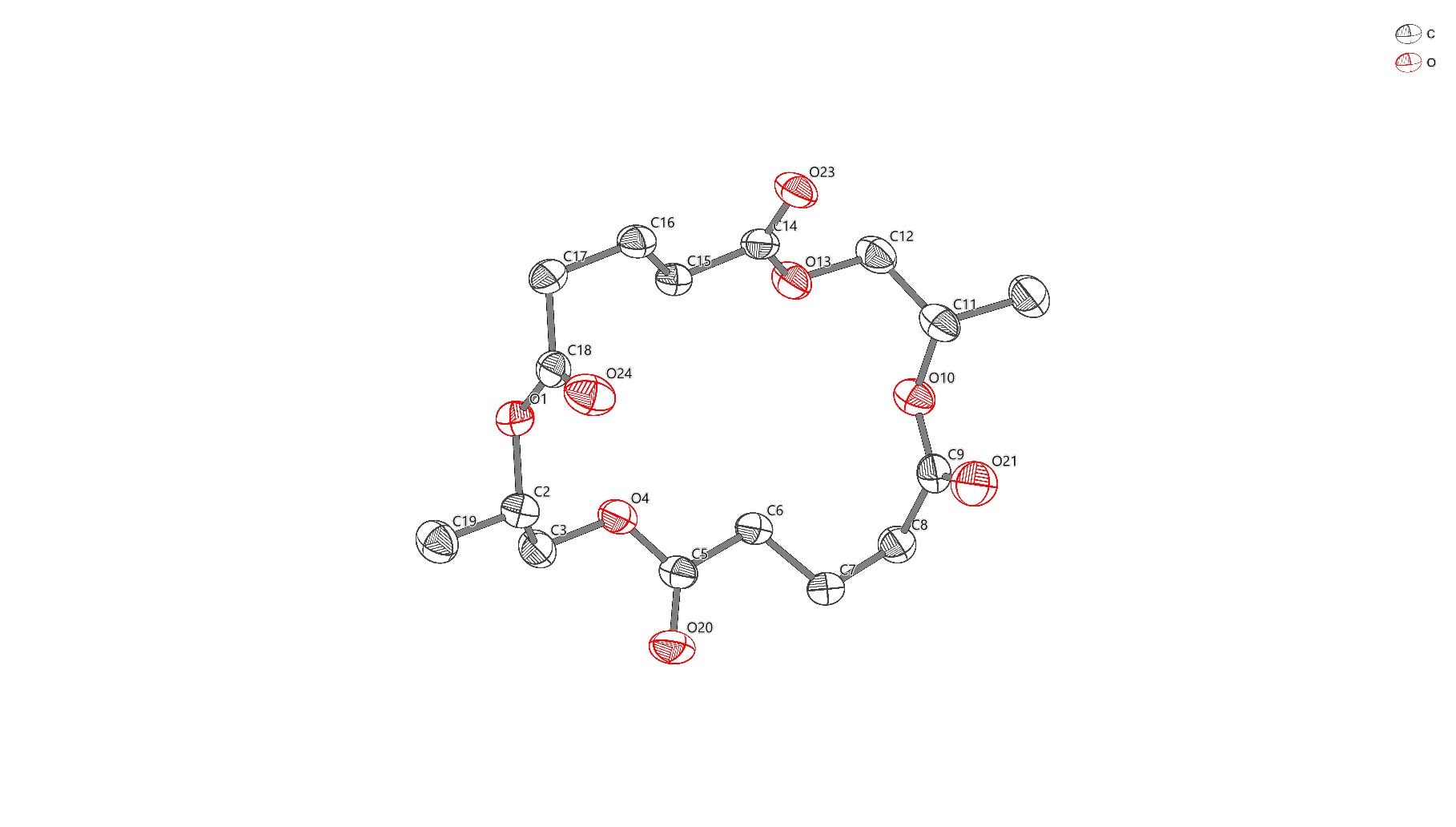


(*S*)

(*S*)

### Figure S23. Molecular structure determined by single crystal X-ray diffraction of the E *cis* (PO-*alt*-GA) tetralactone isomer produced by poly(PO-*alt*-GA) recycling. Depolymerization performed at 230 °C, [Sn(II)Oct_2_]_0_:[poly(PO-*alt*-GA)]_0_ 1:10, 5 mbar pressure. Note that a mixture of *RR* and *SS* isomers (i.e., stereoisomers) are observed but only one is illustrated (S,S). Structure 001rwfk24.

####

###
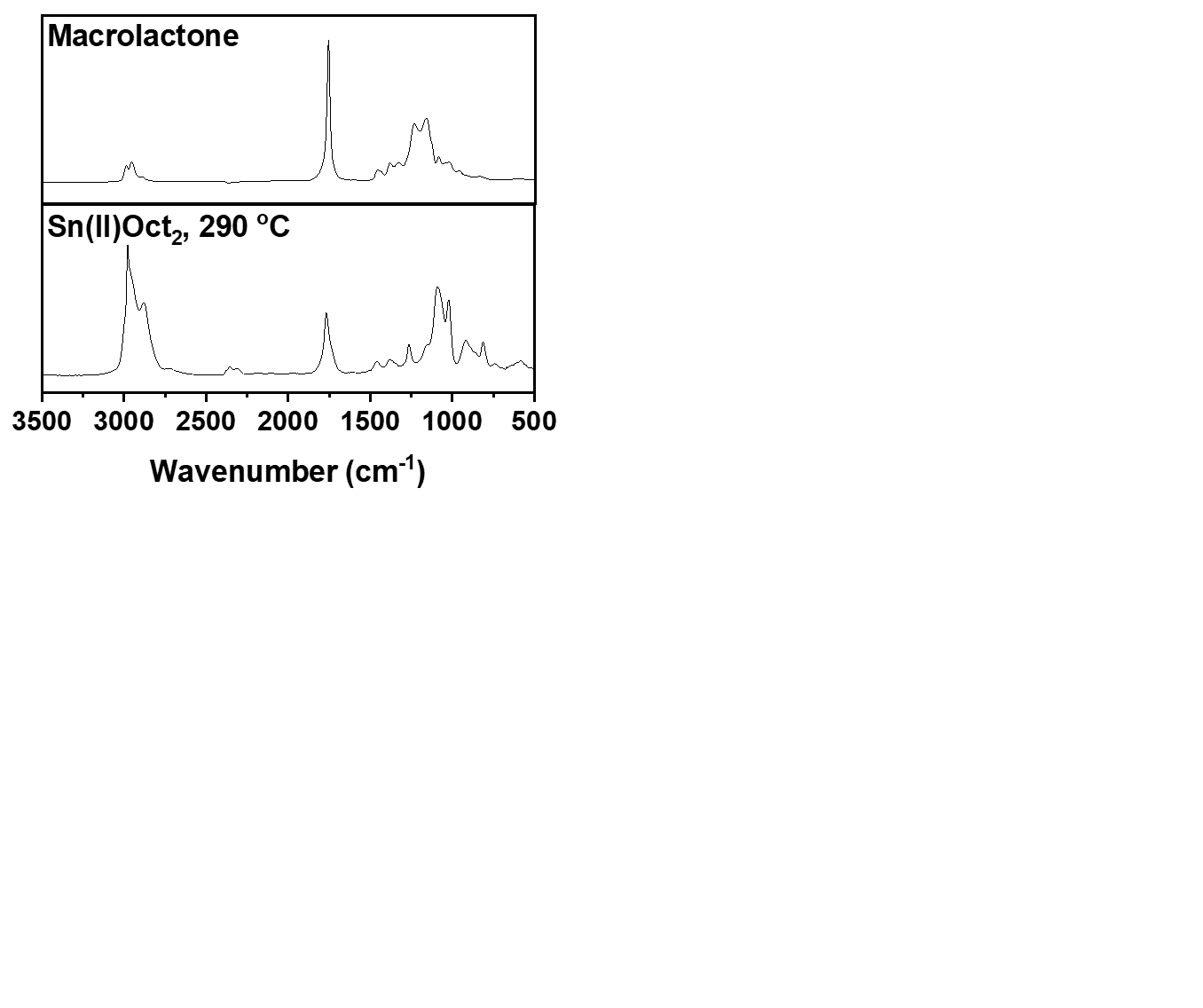
Figure S24. Weight *vs*. Time for isothermal recycling of poly(PO-*alt*-GA) at a) 260 °C and b) 290 °C. Depolymerizations conducted under N_2_ flow rate 25 mL min^-1^, [Sn(II)Oct_2_]_0_:[poly(PO-*alt*-GA)]_0_ 1:500.

### Figure S25. Gas-phase FTIR spectra of (PO-*alt*-GA) tetralactone from the lab-scale recycling of poly(PO-*alt*-GA) (top) and for isothermal depolymerization of poly(PO-*alt*-GA) by Sn(II)Oct_2_ at 290 °C, N_2_ flow rate 25 mL min^-1^, [catalyst]_0_:[poly(PO-*alt*-GA)]_0_ 1:10 (bottom). The high intensity peak in the macrolactone FTIR spectrum at 1754 cm^-1^ can be assigned as the C=O stretch, whilst peaks at 1158cm^-1^ and 1231cm^-1^ correspond to C-O stretches in the ester group.

####

### Figure S26. Weight *vs*. Time for poly(PO-*alt*-GA) held at 230 °C for 10 h, N_2_ flow rate 25 mL min^-1^. This data shows the polymer is stable without addition of catalyst at 230 °C.

####

### Figure S27. Weight *vs*. Time for isothermal recycling of poly(PO-*alt*-GA) at 230 °C. All data is fit to an exponential decay and the rate constant for recycling at each catalyst loading is presented. Depolymerizations conducted under N_2_ flow rate 25 mL min^-1^, [Sn(II)Oct_2_]_0_:[poly(PO-*alt*-GA)]_0_ a) 1:10, b) 1:50, c) 1:100 and d) 1:500.

####

### Figure S28. Ln[weight_t_/weight_0_] *vs*. Time for isothermal recycling of poly(PO-*alt*-GA). All plots shows a linear fit between 5-15 % conversion of polymer. Depolymerizations were conducted under N_2_ flow rate 25 mL min^-1^, [Sn(II)Oct_2_]_0_:[poly(PO-*alt*-GA)]_0_ a) 1:10, b) 1:50, c) 1:100 and d) 1:500.

### Figure S29. a) Weight vs. Time for isothermal recycling of poly(PO-*alt*-GA) at 230 °C with different catalyst loadings. b) Plot of Ln[*k*_obs_] vs. Ln[cat]. The kobs value was determined from the linear fit of the Ln[weight_t_/weight_0_] vs time plot between 5-15 % conversion. Depolymerizations performed under N_2_ flow rate 25 mL min^-1^, 230 °C, [Sn(II)Oct_2_]_0_:[poly(PO-alt-GA)]_0_ 1:10-500.

### Figure S30. ^1^H NMR spectrum (CDCl_3_) for acetyl end-capped poly(PO-*alt*-GA).

####

### Figure S31. ^31^P{^1^H} NMR spectrum (CDCl_3_) for acetal end-capped poly(PO-*alt*-GA) end group test. No peaks relating to hydroxyl end groups are observed.

####

### Figure S32. Weight *vs*. Time for isothermal recycling of acetyl and hydroxyl end-capped poly(PO-*alt*-GA) at 230 °C. Depolymerizations conducted under N_2_ flow rate 25 mL min^-1^, [catalyst]_0_:[poly(PO-*alt*-GA)]_0_ 1:100.

### Figure S33. Weight *vs*. Time for isothermal recycling of poly(PO-*alt*-GA) by Sn(O*^n^*Bu)_2_ and Sn(II)(Oct)_2_ at 230 °C. Depolymerizations conducted under N_2_ flow rate 25 mL min^-1^, [catalyst]_0_:[poly(PO-*alt*-GA)]_0_ 1:100.

### Figure S34. Ln[weight_t_/weight_0_] *vs*. Time for isothermal recycling of poly(PO-*alt*-GA) by Sn(II)(O*^n^*Bu)_2_ at 230 °C. The plot shows a linear fit between 5-15 % conversion of polymer. Depolymerizations conducted under N_2_ flow rate 25 mL min^-1^, [catalyst]_0_:[poly(PO-*alt*-GA)]_0_ 1:100.

####

### Figure S35. Calculated -ΔG° values for the possible cyclic reaction products from the depolymerization of poly(PO-*alt*-GA) relative to propylene oxide and glutaric anhydride.

### Table S3. Calculated -ΔG° values for the possible cyclic reaction products from the depolymerization of poly(PO-*alt*-GA).

|  | ΔG° (kcal) | ΔH° (kcal) | ΔG° - ΔG°_PO+GA_ (kcal)*^[a]^* | ΔH° - ΔH°_PO+GA_ (kcal)*^[a]^* |
| --- | --- | --- | --- | --- |
| PO | -121134.1154 | -121114.2387 | - | - |
| GA | -263389.6273 | -263364.7758 | - | - |
| PO+GA | -384523.74273 | -384479.01453 | 0 | 0 |
| Dilactone | -384534.25335 | -384503.24795 | -10.5 | -24.2 |
| E-trans tetralactone | -769090.739 | -769040.4562 | -21.6 | -41.2 |
| E-cis tetralactone | -769081.1439 | -769030.2813 | -16.8 | -36.1 |
| Z-cis tetralactone | -769099.8102 | -769047.338 | -26.2 | -70.9 |
| Z-trans tetralactone | -769079.5005 | -769029.9782 | -16.0 | -36.0 |
| *SSS* HHT hexalactone | -1153633.138 | -1153563.375 | -20.6 | -42.1 |
| *SSR* HHH hexalactone | -1153634.579 | -1153566.176 | -21.1 | -43.0 |
| *SRR* HHT hexalactone | -1153635.229 | -1153566.76 | -21.3 | -43.2 |
| *SSR* HHT hexalactone | -1153629.484 | -1153560.596 | -19.4 | -41.2 |
| *SSS* HHH hexalactone | -1153629.723 | -1153560.453 | -19.5 | -41.1 |
| *RSR* HHT hexalactone | -1153628.339 | -1153558.986 | -19.0 | -40.6 |

*^[a]^*Calculated per PO-alt-GA repeat unit relative the formation of the epoxide and anhydride.

### Table S4. Screening of various catalysts for the ROP of the (PO-*alt*-GA) tetralactone.*^[a]^*

| Catalyst | [catalyst]_0_:[benzyl alcohol]_0_:[tetralactone]_0_ | Temperature (°C) | Conversion*^[b]^* |
| --- | --- | --- | --- |
| P_4_-*^t^*Bu | 1:1:50 | 100 | No reaction |
| Ti(O*^i^*Pr)_4_ | 1:1:100 | 100 | No reaction |
| Ti(O*^i^*Pr)_4_ | 1:1:100 | 150 | No reaction |
| Y(N(Si(CH_3_)_3_)_2_)_3_ | 1:1:50 | 150 | No reaction |
| Y(III)Oct_3_ | 1:1:50 | 150 | 31 % conversion after 20 h |
| Sn(II)Oct_2_ | 1:1:50 | 150 | >60 % conversion after 20 h |

*^[a]^*Polymerization performed neat under given [catalyst]_0_:[benzyl alcohol]_0_:[tetralactone]_0_ ratios. *^[b]^*Conversion determined from the relative integrals of the peaks at 4.38 – 4.21 ppm, corresponding to H_e’_ in the (PO-*alt*-GA) tetralactone, to 4.23 – 4.13 ppm, corresponding to H_e_ in poly(PO-*alt*-GA), in the ^1^H NMR spectrum of the reaction mixture after 20 h.

### Figure S36. ^1^H NMR spectrum (CDCl_3_) of the recycled poly(PO-*alt*-GA) after 90 h. ROP of (PO-*alt*-GA) tetralactone performed neat at 150 °C, [Sn(II)Oct_2_]_0_:[benzyl alcohol]_0_:[tetralactone]_0_ 1:1:50.

### Figure S37. SEC trace of the recycled poly(PO-*alt*-GA) after 90 h and SEC trace of virgin polymer. ROP of (PO-*alt*-GA) tetralactone performed neat at 150 °C, [Sn(II)Oct_2_]_0_:[benzyl alcohol]_0_:[tetralactone]_0_ 1:1:50.

### Figure S38. Van’t Hoff analysis of the macrolactone polymerization. ROP of (PO-*alt*-GA) tetralactone performed neat at 140 °C, 150 °C and 160 °C, [Sn(II)Oct_2_]_0_:[benzyl alcohol]_0_:[tetralactone]_0_ 1:1:50. [M]_eq_ determined by ^1^H NMR.

####

### Figure S39. Weight *vs*. Temperature for recycling of poly(PO-*alt*-SA) by Sn(II)Oct_2_. Depolymerizations conducted under N_2_ flow rate 25 mL min^-1^, heating rate 2 °C min^-1^, [Sn(II)Oct_2_]_0_:[poly(PO-*alt*-SA)]_0_ 1:500.

####

### Figure S40. Weight *vs*. Temperature for recycling of poly(BO-*alt*-GA). Depolymerizations conducted under N_2_ flow rate 25 mL min^-1^, heating rate 2 °C min^-1^, [Sn(II)Oct_2_]_0_:[poly(BO-*alt*-GA)]_0_ 1:500.

####

### Figure S41. Weight *vs*. Time for poly(PO-*alt*-SA) held at 230 °C for 10 h, N_2_ flow rate 25 mL min^-1^.

####

### Figure S42. Weight *vs*. Time for poly(BO-*alt*-GA) held at 230 °C for 10 h, N_2_ flow rate 25 mL min^-1^.

####

### Figure S43. a) Weight *vs*. Time for isothermal recycling of poly(PO-*alt*-SA) at 230 °C. The data is fit to an exponential decay and the rate constant for recycling is shown. b) Ln[weight_t_/weight_0_] *vs*. Time for isothermal recycling of poly(PO-*alt*-SA) by at 230 °C. The plot shows a linear fit between 5-15 % conversion of the polymer. Depolymerization conducted under N_2_ flow rate 25 mL min^-1^, [Sn(II)Oct_2_]_0_:[poly(PO-*alt*-SA)]_0_ 1:10.

### Figure S44. a) Weight *vs*. Time for isothermal recycling of poly(BO-*alt*-GA) at 230 °C. The data is fit to an exponential decay and the rate constant for recycling is shown. b) Ln[weight_t_/weight_0_] *vs*. Time for isothermal recycling of poly(BO-*alt*-GA) at 230 °C. The plot shows a linear fit between 5-15% conversion of the polymer. Depolymerization conducted under N_2_ flow rate 25 mL min^-1^, [catalyst]_0_:[poly(BO-*alt*-GA)]_0_ 1:10.

### Figure S45. ^1^H NMR spectrum (CDCl_3_) of the crude product from the lab-scale recycling of poly(PO-*alt*-SA). Depolymerization performed at 210 °C, 20 mbar [Sn(II)Oct_2_]_0_:[ poly(PO-*alt*-SA)]_0_ 1:10. Splitting of the peaks at d’, c’, b’ and a’ are due to the 4 different regio- and stereo- isomers of the tetralactone.

### Figure S46. ^13^C NMR spectrum (CDCl_3_) of the crude product from the lab-scale recycling of poly(PO-*alt*-SA). Depolymerization performed at 210 °C, 20 mbar [Sn(II)Oct_2_]_0_:[ poly(PO-*alt*-SA)]_0_ 1:10.

### Figure S47. GC of the crude product from the lab-scale recycling of poly(PO-*alt*-SA). All peaks between 20.61 – 20.86 min corresponding to *m/z* = 316.29. This is a match to the theoretical mass of the proposed 16-membered ring (*m/z*_theo_ = 316.12). Selectivity for the tetralactone by GC is >99%. Depolymerization performed at 210 °C, 20 mbar [Sn(II)Oct_2_]_0_:[ poly(PO-*alt*-SA)]_0_ 1:10.

### Figure S48. ^1^H NMR spectrum (CDCl_3_) of the crude product from the lab-scale recycling of poly(BO-*alt*-GA). Depolymerization performed at 230 °C, 4 mbar, [Sn(II)Oct_2_]_0_:[ poly(BO-*alt*-GA)]_0_ 1:10. Selectivity for tetralactone = 80 % by ^1^H NMR spectroscopy. The resonances at δ 2.74 and 2.09 ppm are attributed to trace glutaric anhydride and the resonances at δ 7.36, 4.70 and 2.99 ppm are attributed to trace benzyl alcohol.

### Figure S49. ^13^C NMR spectrum (CDCl_3_) of the crude product from the lab-scale recycling of poly(BO-*alt*-GA). Depolymerization performed at 230 °C, 4 mbar [Sn(II)Oct_2_]_0_:[ poly(BO-*alt*-GA)]_0_ 1:10.

### Figure S50. GC of the crude product from the lab-scale recycling of poly(BO-*alt*-GA). Peaks at 23.81 and 23.85 min corresponding to *m/z* = 372.17. This is a match to the theoretical mass of the proposed 18-membered ring (*m/z*_theo_ = 372.18). Selectivity for the tetralactone by GC is 74%. Depolymerization performed at 230 °C, 4 mbar [Sn(II)Oct_2_]_0_:[ poly(BO-*alt*-GA)]_0_ 1:10.

### Figure S51. Na^+^ mass spectrum of the (PO-*alt*-SA) tetralactone collected from the lab-scale recycling of poly(PO-*alt*-SA) (top spectrum) and theoretical Na^+^ mass spectrum of the (PO-*alt*-SA) tetralactone (bottom). Depolymerization performed at 210 °C, 20 mbar [Sn(II)Oct_2_]_0_:[ poly(PO-*alt*-SA)]_0_ 1:10.


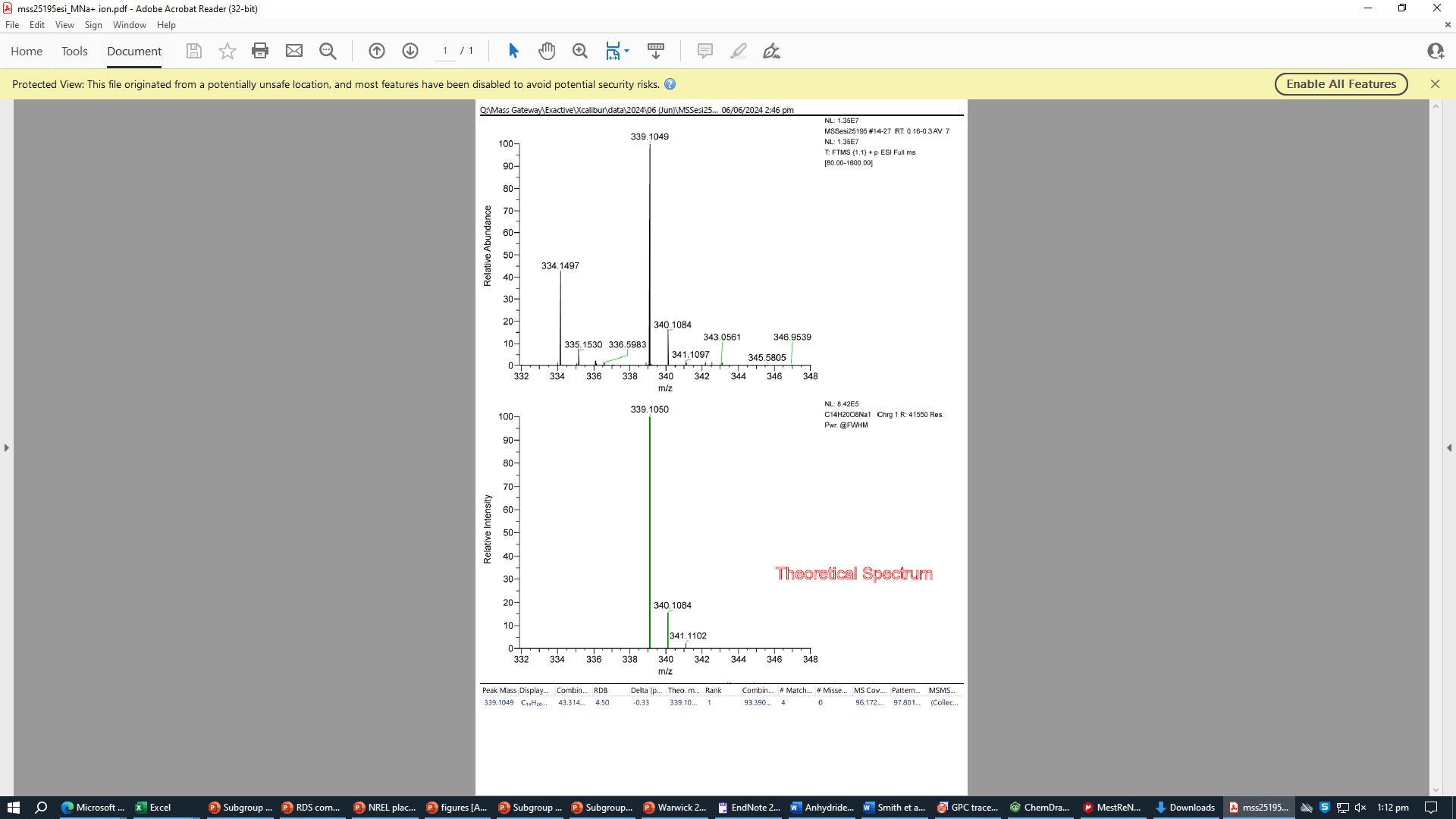


### Figure S52. Na^+^ mass spectrum of the (BO-*alt*-GA) tetralactone collected from the lab-scale recycling of poly(BO-*alt*-GA) (top spectrum) and theoretical Na^+^ mass spectrum of the (BO-*alt*-GA) tetralactone (bottom). Depolymerization performed at 230 °C, 4 mbar [Sn(II)Oct_2_]_0_:[ poly(BO-*alt*-GA)]_0_ 1:10.


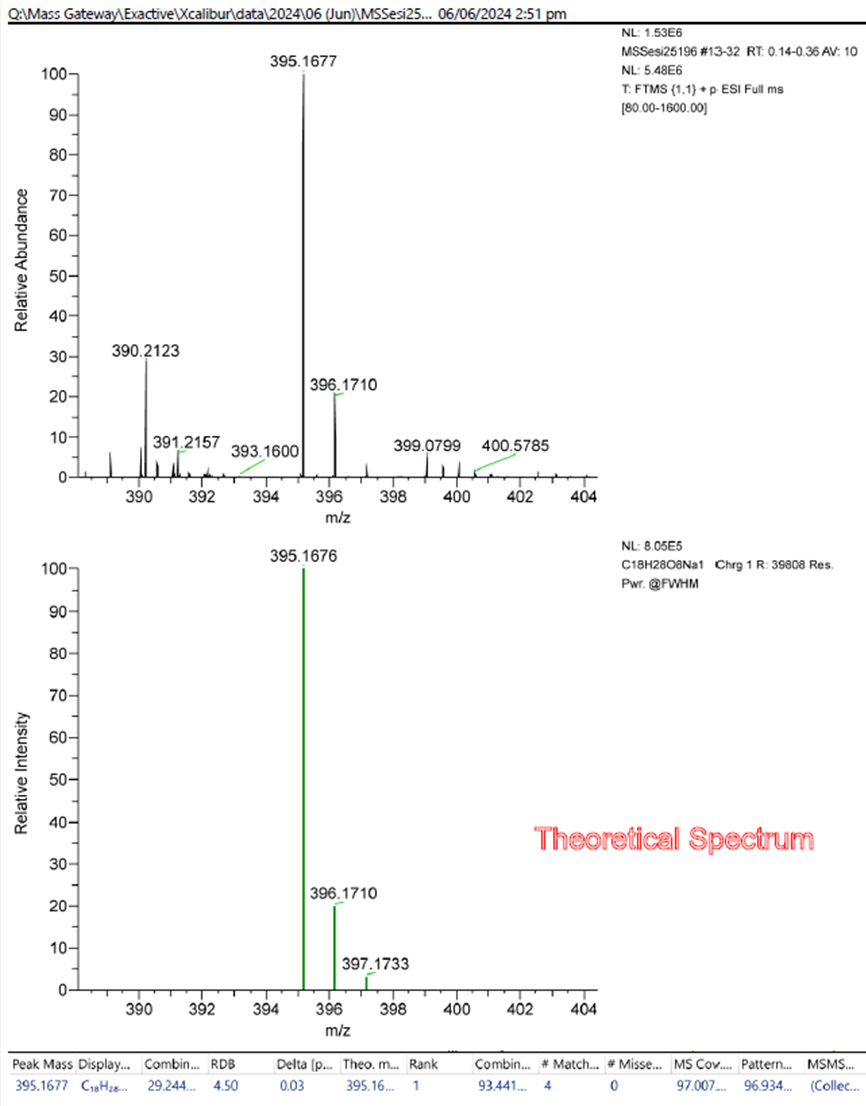


**F**igure S53. Molecular structure determined by single crystal X-ray diffraction of the (BO-*alt*-GA) tetralactone collected from the large scale recycling of poly(BO-*alt*-GA). Depolymerization performed at 230 °C, 4 mbar [Sn(II)Oct_2_]_0_:[ poly(BO-*alt*-GA)]_0_ 1:10 using Kugelrohr apparatus. Structure 007rwfk24.


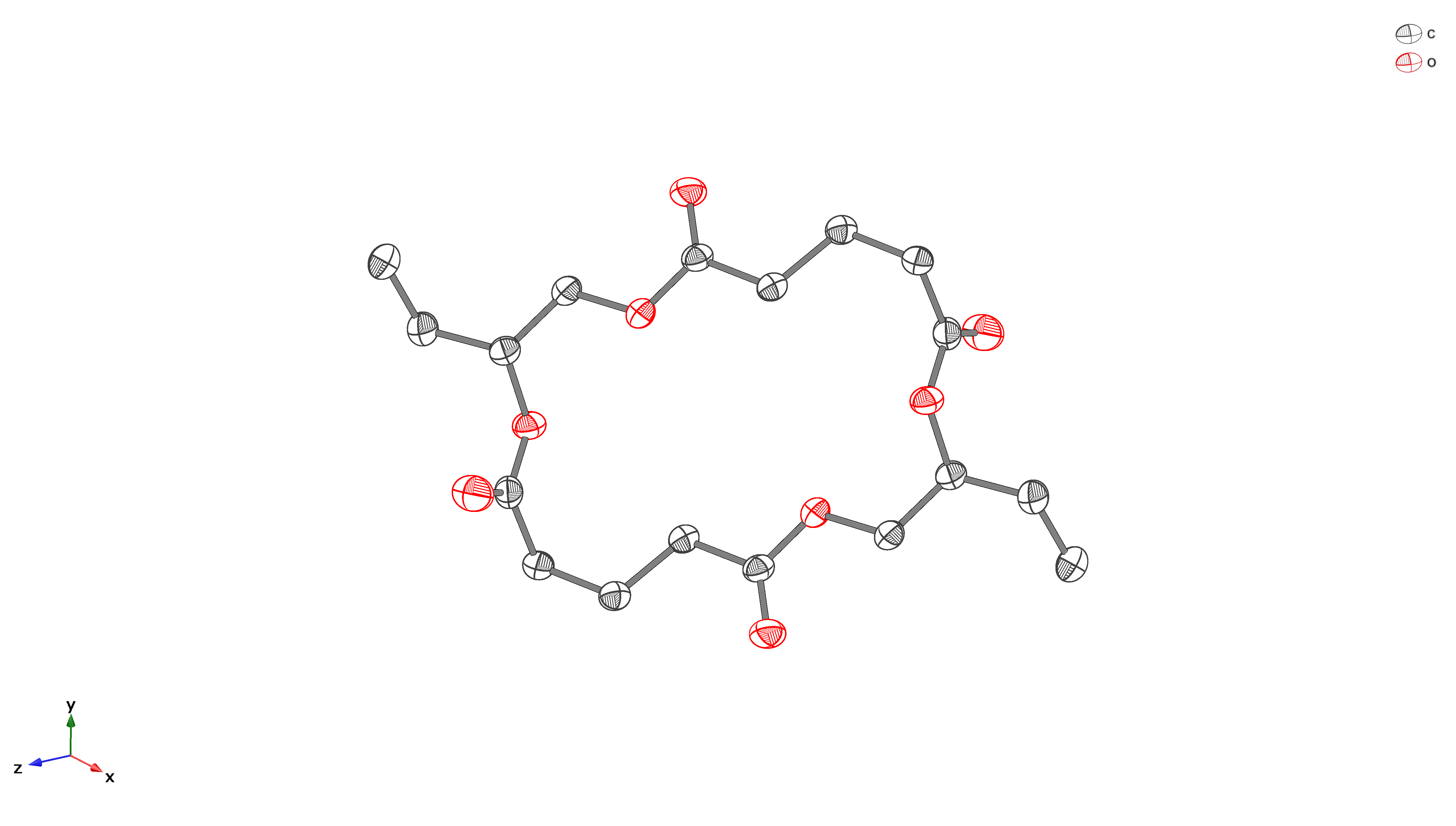

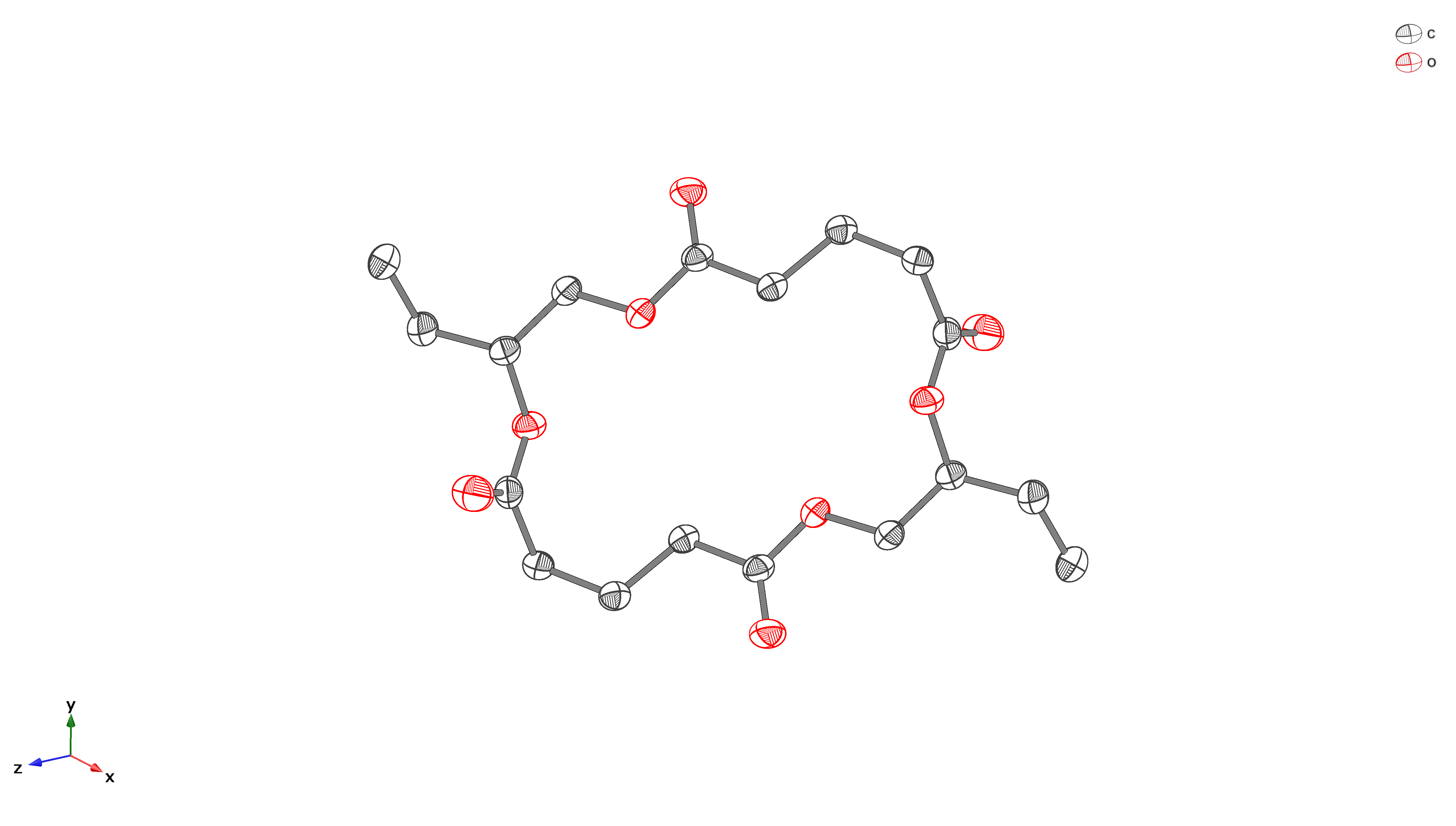


*(S)*

*(R)*

## Discussion of Regio- and Stereo- Selectivity

Both the starting polymer and the macrolactones contain all possible regioisomers and in approximately equal amounts. This has been determined through use of ^1^H NMR, quantitative ^13^C NMR and decoupled ^1^H NMR spectroscopy (Figs. S4, S14, S18).

The quantitative ^13^C{^1^H} NMR spectrum of the tetralactone displays 8 carbonyl signals at approximately δ 172 ppm, together with 4 signals each at δ 68 and 66 ppm assigned to the CH_2_ c’ carbon and CH e’ carbon atoms, respectively (Figure S14). The integrals of these methylene/methyne signals are in a 1:1:1:1 ratio, indicating that all isomers are present in equal amounts.

The quantitative ^13^C{^1^H} NMR spectrum of the starting polymer also revealed 4 signals: at ~ 172.6 (2 signals) and 172.3 (2 signals) ppm. These are assigned to the regio- (head to head or head to tail linkages) and stereoisomers (Figure S4). The integration of these signals was approximately 1:1:1:1 which suggests the polymer is regio-random.

The ^1^H{^1^H} NMR spectrum of the tetralactone shows 4 resonances assigned to methyl groups (1.24 ppm). This data also suggests all 4 regioisomers of the tetralactone are present. Attempts to integrate these resonances were complicated by signal over-lap (Figure S18).

The decoupled ^1^H{^1^H} NMR spectrum of the polymer was also examined for the same methyl resonance, but decoupling was inconclusive due to signal broadening.

The polymer chain-end group was analysed, after reaction with a phorphoryl chloride reagent (which reacts with hydroxyl end-groups) using ^31^P {^1^H} NMR spectroscopy (Figure S5). The spectrum shows signals consistent with both primary (1°) and secondary (2°) alcohol end groups. This data suggests that both (epoxide ring-opened) regioisomers are present at the chain end.

Both the polymer and the tetralactone show evidence for a lack of regioselectivity or stereoselectivity. Thus, we believe that the regio/stereo-random polymer results in the formation of all possible isomers of the macrolactone.

## Crystallographic Data

Crystalline samples were mounted on a MiTeGen Micromount and cooled to 150 K, with dry nitrogen flow, using an Oxford Cryostream.^47^ Data was collected with an Oxford Diffraction Supernova diffractometer using Cu Kα (λ = 1.5417 Å) radiation. The resulting reflection data was processed with CrysAlis Pro.^48-50^ Structures were solved using the SHELXT program and least-square refined using the SHELXL program within the Olex2 system suite.^49-51^

**Table 1. Experimental details**

| **Crystal data** | | | |
| --- | --- | --- | --- |
| Local Code | 012rwfk23 | 001rwfk24 | 007rwfk24 |
| CCDC Deposition Number | 2379205 | 2379206 | 2379207 |
| Name | (PO-*alt*-GA) E *trans* | (PO-*alt*-GA) E *cis* | (BO-*alt*-GA) E *trans* |
| Chemical formula | C_16_H_24_O_8_ | C_16_H_24_O_8_ | 2(C_9_H_14_O_4_) |
| *M*_r_ | 344.35 | 344.35 | 372.40 |
| Crystal system, space group | Orthorhombic, *Pna*2_1_ | Triclinic, *P*¯1 | Monoclinic, *P*2_1_/*c* |
| Temperature (K) | 150 | 150 | 150 |
| *a*, *b*, *c* (Å) | 11.9233 (4), 18.5283 (5), 7.7156 (2) | 8.9873 (5), 10.2465 (6), 10.5893 (6) | 13.7288 (3), 7.6946 (2), 18.5889 (5) |
| a, b, g (°) | 90, 90, 90 | 83.881 (5), 77.878 (5), 64.723 (6) | 90, 106.292 (3), 90 |
| *V* (Å^3^) | 1704.52 (9) | 861.97 (10) | 1884.83 (9) |
| *Z* | 4 | 2 | 4 |
| m (mm^-1^) | 0.91 | 0.90 | 0.86 |
| Crystal size (mm) | 0.53 × 0.26 × 0.21 | 0.13 × 0.08 × 0.05 | 0.28 × 0.23 × 0.14 |
| **Data collection** | | | |
| Diffractometer | SuperNova, Dual, Cu at home/near, Atlas | | |
| Absorption correction | Gaussian  *CrysAlis PRO* 1.171.40.53 (Rigaku Oxford Diffraction, 2019) Numerical absorption correction based on gaussian integration over a multifaceted crystal model Empirical absorption correction using spherical harmonics, implemented in SCALE3 ABSPACK scaling algorithm. | Multi-scan  *CrysAlis PRO* 1.171.40.53 (Rigaku Oxford Diffraction, 2019) Empirical absorption correction using spherical harmonics, implemented in SCALE3 ABSPACK scaling algorithm. | Gaussian  *CrysAlis PRO* 1.171.40.53 (Rigaku Oxford Diffraction, 2019) Numerical absorption correction based on gaussian integration over a multifaceted crystal model Empirical absorption correction using spherical harmonics, implemented in SCALE3 ABSPACK scaling algorithm. |
| *T*_min_, *T*_max_ | 0.316, 1.000 | 0.882, 1.000 | 0.413, 1.000 |
| No. of measured, independent and  observed [*I* > 2s(*I*)] reflections | 16408, 2908, 2819 | 9752, 3571, 2953 | 19856, 3913, 3633 |
| *R*_int_ | 0.038 | 0.025 | 0.023 |
| (sin q/l)_max_ (Å^-1^) | 0.630 | 0.631 | 0.630 |
| Refinement | | | |
| *R*[*F*^2^ > 2s(*F*^2^)], *wR*(*F*^2^), *S* | 0.037, 0.097, 1.05 | 0.043, 0.116, 1.06 | 0.042, 0.116, 1.04 |
| No. of reflections | 2908 | 3571 | 3913 |
| No. of parameters | 219 | 219 | 237 |
| No. of restraints | 1 | 0 | 0 |
| Dρ_max_, Dρ_min_ (e Å^-3^) | 0.27, -0.19 | 0.48, -0.19 | 0.39, -0.25 |
| Absolute structure | Flack x determined using 911 quotients [(I+)-(I-)]/[(I+)+(I-)] (Parsons, Flack and Wagner, Acta Cryst. B69 (2013) 249-259). | – | – |
| Absolute structure parameter | -0.10 (16) | – | – |

## References

(37) Flynn, J. H.; Wall, L. A. A quick, direct method for the determination of activation energy from thermogravimetric data. *J. Polym. Sci., Part B: Polym. Lett.* **1966**, *4* (5), 323-328.

(43) Li, C.; Sablong, R. J.; Van Benthem, R. A. T. M.; Koning, C. E. Unique Base-Initiated Depolymerization of Limonene-Derived Polycarbonates. *ACS Macro Lett.* **2017**, *6* (7), 684-688. DOI: 10.1021/acsmacrolett.7b00310.

(44) *Gaussian 16 Rev. C.01*; Wallingford, CT, 2016.

(45) Chai, J.-D.; Head-Gordon, M. Long-range corrected hybrid density functionals with damped atom–atom dispersion corrections. *Phys. Chem. Chem. Phys.* **2008**, *10* (44), 6615-6620.

(46) Chai, J.-D.; Head-Gordon, M. Optimal operators for Hartree–Fock exchange from long-range corrected hybrid density functionals. *Chem. Phys. Lett.* **2008**, *467* (1-3), 176-178.

(47) Cosier, J. t.; Glazer, A. A nitrogen-gas-stream cryostat for general X-ray diffraction studies. *J. Appl. Crystallogr.* **1986**, *19* (2), 105-107.

(48) CrysAlis Pro. v1.171. 40.53 a, Oxford Diffraction /Agilent Technologies UK Ltd, Yarnton, England, 2019.

(49) Dolomanov, O. V.; Bourhis, L. J.; Gildea, R. J.; Howard, J. A.; Puschmann, H. OLEX2: a complete structure solution, refinement and analysis program. *J. Appl. Crystallogr.* **2009**, *42* (2), 339-341.

(50) Sheldrick, G. M. *SHELXT-2018/2, Göttingen, Germany*. Vol. 2. SHELXT-2018, 2018.

(51) Sheldrick, G. M. Crystal structure refinement with SHELXL. *Acta Crystallogr., Sec. C:* *Struct. Chem*. **2015**, *71* (1), 3-8.
